# Clinical Performance of the Novel GenMark Dx ePlex Blood Culture ID Gram-Positive Panel

**DOI:** 10.1128/JCM.01730-19

**Published:** 2020-03-25

**Authors:** Karen C. Carroll, Jennifer L. Reid, Adam Thornberg, Natalie N. Whitfield, Deirdre Trainor, Shawna Lewis, Teresa Wakefield, Thomas E. Davis, Keisha G. Church, Linoj Samuel, Ray Mills, Patricia Jim, Stephen Young, Frederick S. Nolte

**Affiliations:** a Division of Medical Microbiology, Department of Pathology, Johns Hopkins Medical Institutions, Baltimore, Maryland, USA; b GenMark Diagnostics Inc., Carlsbad, California, USA; c Indiana University School of Medicine, Indianapolis, Indiana, USA; d Medical University of South Carolina, Charleston, South Carolina, USA; e Henry Ford Health System, Detroit, Michigan, USA; f Tricore Reference Laboratories, Albuquerque, New Mexico, USA; bioMérieux

**Keywords:** blood culture, eSensor technology, Gram-positive bacteria, identification, multiplex nucleic acid test, resistance marker

## Abstract

Rapid identification from positive blood cultures is standard of care (SOC) in many clinical microbiology laboratories. The GenMark Dx ePlex Blood Culture Identification Gram-Positive (BCID-GP) Panel is a multiplex nucleic acid amplification assay based on competitive DNA hybridization and electrochemical detection using eSensor technology. This multicenter study compared the investigational-use-only (IUO) BCID-GP Panel to other methods of identification of 20 Gram-positive bacteria, four antimicrobial resistance genes, and both Pan *Candida* and Pan Gram-Negative targets that are unique to the BCID-GP Panel. Ten microbiology laboratories throughout the United States collected residual, deidentified positive blood culture samples for analysis. Five laboratories tested both clinical and contrived samples with the BCID-GP Panel. Comparator identification methods included each laboratory’s SOC, which included matrix-assisted laser desorption ionization–time of flight mass spectrometry (MALDI-TOF MS) and automated identification systems as well as targeted PCR/analytically validated real-time PCR (qPCR) with bidirectional sequencing. A total of 2,342 evaluable samples (1,777 clinical and 565 contrived) were tested with the BCID-GP Panel. The overall sample accuracy for on-panel organisms was 89% before resolution of discordant results. For pathogenic Gram-positive targets (Bacillus cereus group, Enterococcus spp., Enterococcus faecalis, Enterococcus faecium, Staphylococcus spp., Staphylococcus aureus, Staphylococcus epidermidis, Staphylococcus lugdunensis, Listeria spp., Listeria monocytogenes, Streptococcus spp., Streptococcus agalactiae, Streptococcus anginosus group, Streptococcus pneumoniae, and Streptococcus pyogenes), positive percent agreement (PPA) and negative percent agreement (NPA) ranged from 93.1% to 100% and 98.8% to 100%, respectively. For contamination rule-out targets (Bacillus subtilis group, *Corynebacterium*, Cutibacterium acnes, *Lactobacillus*, and *Micrococcus*), PPA and NPA ranged from 84.5% to 100% and 99.9% to 100%, respectively. Positive percent agreement and NPA for the Pan *Candida* and Pan Gram-Negative targets were 92.4% and 95.7% for the former and 99.9% and 99.6% for the latter. The PPAs for resistance markers were as follows: *mecA*, 97.2%; *mecC*, 100%; *vanA*, 96.8%; and *vanB*, 100%. Negative percent agreement ranged from 96.6% to 100%. In conclusion, the ePlex BCID-GP Panel compares favorably to SOC and targeted molecular methods for the identification of 20 Gram-positive pathogens and four antimicrobial resistance genes in positive blood culture bottles. This panel detects a broad range of pathogens and mixed infections with yeast and Gram-negative organisms from the same positive blood culture bottle.

## Abstract (precis)

Rapid identification from positive blood cultures is standard of care (SOC) in many clinical microbiology laboratories. The GenMark Dx ePlex Blood Culture Identification Gram-Positive (BCID-GP) Panel is a multiplex nucleic acid amplification assay based on competitive DNA hybridization and electrochemical detection using eSensor technology. This multicenter study compared the investigational-use-only (IUO) BCID-GP Panel to other methods of identification of 20 Gram-positive bacteria, four antimicrobial resistance genes, and both Pan *Candida* and Pan Gram-Negative targets that are unique to the BCID-GP Panel.

## INTRODUCTION

In 2016, septicemia was ranked as the 11th leading cause of death in the United States (1). While outcomes for critically ill patients have improved, worldwide trends indicate increasing incidences of bacteremia and candidemia (2–5). In addition, reports indicate that the number of intensive care unit admissions related to community-acquired bacteremia have increased, likely related to the aging population and associated comorbidities (2). It was estimated that 19 to 28% of bloodstream infections in North America were nosocomially acquired (6). Regardless of whether infections are acquired in the community or the hospital, mortality remains high. Poor outcomes, including death, related to bloodstream infections correlate with delayed administration of active antimicrobial treatment (7–10).

Rapid detection of the causative organism in positive blood cultures, especially when combined with antimicrobial stewardship, reduces the interval between recognition of bacteremia and appropriate therapy by 18 to 24 h or longer (11–14). In a systematic review by Timbrook et al., it was determined that when the use of rapid molecular diagnostic methods in the identification of the microorganism causing bloodstream infections was combined with antimicrobial stewardship interventions, mortality risk was significantly lower than with conventional microbiology methods (15), prompting the authors to recommend their use as standard of care. Several pathogen-specific assays, broader multiplex panel tests applying nucleic acid amplification and nonamplified array technologies, and matrix-assisted laser desorption ionization–time of flight mass spectrometry (MALDI-TOF MS) have been routinely incorporated into many laboratory workflows in the United States (11–16). Many of these assays are limited by the number of pathogens they can detect, and almost all of them have difficulty with differentiation of species in polymicrobial bacteremia (17–19). Differentiation of species is an important issue since up to 8 to 12% of bloodstream infections are reported to be polymicrobial (4, 8).

The GenMark Dx ePlex blood culture identification Gram-positive (BCID-GP) Panel is an FDA-cleared qualitative multiplex nucleic acid amplification assay intended for use on the GenMark Dx ePlex instrument that detects 20 Gram-positive species or groups and *mecA*, *mecC*, *vanA*, and *vanB* resistance genes. In addition, the panels contain Pan Gram-Negative and Pan *Candida* probes for extended coverage outside of the Gram-positive organisms. The goal of this multicenter study was to evaluate the clinical performance of the GenMark Dx ePlex investigational-use-only (IUO) BCID-GP Panel compared to conventional methods for identification and antimicrobial resistance gene detection for common Gram-positive organisms recovered from positive blood cultures.

## MATERIALS AND METHODS

### Study population.

The study population included patients of all ages and genders with positive blood cultures collected for standard patient care and diagnosis at 10 geographically diverse clinical sites located in the United States from the following nine cities: Albuquerque, NM; Baltimore, MD; Charleston, SC; Danville, PA; Detroit, MI (two sites); Harvey, IL; Indianapolis, IN; Milwaukee, WI; and San Diego, CA.

### Specimen collection.

A total of 719 samples with positive Gram stain results were prospectively collected; 400 samples were collected from June 2014 through July 2016 from three sites and frozen for future testing, and 319 samples were collected from January through March 2018 from five sites and tested fresh. The majority of samples were collected from subjects at hospitals or medical centers. To supplement the results of the prospective collection, 586 samples with positive Gram stain results for lower-prevalence Gram-positive organisms were retrospectively collected from 10 sites. Additionally, 566 samples were contrived for Gram-positive organisms with very low prevalence. For evaluation of the Pan targets only, 484 retrospective samples with Gram-negative or fungal Gram stain results were included.

Contrived samples were prepared by aseptically injecting 3 to 10 ml of human whole blood (Bioreclamation, Westbury, NY) into a BD Bactec blood culture bottle (Plus Aerobic/F, Plus Anaerobic/F, Lytic/10 Anaerobic/F, or Peds Plus/F). Table S1 in the supplemental material contains a list of organisms used for the contrived samples. The 27 *Micrococcus* samples were contrived using quantified glycerol stocks spiked into incubated blood. For the remaining samples, the bottles were then inoculated with a colony or colonies from a pure culture. The bacterial suspension was prepared by using a loop to collect fresh culture from an agar plate and suspending it in saline to approximately a 0.5 McFarland standard via reading the optical desity at 600 nm (OD_600_) (0.5 McFarland is approximately 1.5 × 10^8^ CFU/ml). This bacterial suspension was diluted either 1:100 (*n* = 483), 1:1,000 (*n* = 27), or 1:10,000 (*n* = 29), and then either 100 μl (*n* = 499) or 1 ml (*n* = 40) was used to inoculate a Bactec bottle containing blood. The 1-ml inoculum was used for some of the *Bacillus* and Cutibacterium acnes replicates. The inoculum volumes were determined based on successful growth and time to detection in preliminary studies. The CFU added to the bottles ranged from ∼1 × 10^3^ to 2 × 10^6^. The time to detection varied from 7 h to 5 days in 97% of the samples and was up to 10 days for the remaining samples. Of note, the organisms that required more than 5 days were *Bacillus* spp., *Lactobacillus* spp., *Corynebacterium* spp., and C. acnes.

### Overall study design and conduct.

Prospectively and retrospectively collected samples were cultured and tested as ordered, per the sites’ standard operating procedures. All samples were deidentified, residual positive blood culture samples provided after the standard-of-care (SOC) testing was completed. The study was performed using two protocols, one for collection only and a second for collection and testing; both were approved by a central institutional review board (IRB) (Quorum, Seattle, WA) and/or the site’s IRB.

Samples were tested at one of five clinical sites with the GenMark Dx ePlex IUO Blood Culture Identification Gram-Positive (BCID-GP) Panel. Investigational results were compared to the sites’ SOC procedures for organism identification and sequencing for antimicrobial resistance genes (ARGs).

### GenMark Dx ePlex BCID-GP Panel testing.

The BCID-GP Panel was tested within 12 h of positivity as instructed by the manufacturer using the materials in the kit. Briefly, after the blood culture bottle was inverted several times to mix, 50 μl was removed and added to the BCID-GP Panel cartridge through the sample loading port and the cap was depressed to close the port. Each cartridge was barcoded according to the manufacturer’s instructions, scanned at the ePlex instrument, and inserted into an available bay. Upon completion of the assay run, the ePlex instrument ejected the cartridge for disposal and a BCID-GP Panel report was generated.

The BCID-GP Panel runs on the ePlex instrument, which automates all aspects of nucleic acid testing, including extraction, amplification, and detection, combining electrowetting and GenMark’s eSensor technology in a single-use cartridge. eSensor technology is based on the principles of competitive DNA hybridization and electrochemical detection as previously described (20).

The BCID-GP Panel identifies 20 Gram-positive bacteria and four antimicrobial resistance genes (Table 1). It should be noted that if there are multiple Gram-positive cocci in a sample that is also positive for a resistance marker, there is no way to link the gene to the detected species. This would likely most commonly occur with *mecA* when the sample contains Staphylococcus aureus and one or more other staphylococci. The BCID-GP Panel also contains two targets to detect non-Gram-positive organisms using a Pan target approach. The Pan Gram-Negative target detects up to 95% of Gram-negative bacteria, including (but not limited to) *Enterobacterales*, Acinetobacter, *Pseudomonas*, *Bacteroides*, Stenotrophomonas maltophilia, *Serratia*, and *Neisseria*. Four key *Candida* species (Candida albicans, C. glabrata, C. krusei, and C. parapsilosis) are detected by the Pan *Candida* target. Like other commercial syndromic panel tests for use on positive blood culture bottles, this panel is designed to be a useful, rapid adjunct for the diagnosis of bloodstream infections.

**TABLE 1:** Targets detected by the ePlex BCID-GP Panel

Type of target	Organism(s) or gene
Bacterium	Bacillus cereus group^*a*^
	Bacillus subtilis group^*b*^
	*Corynebacterium*
	*Enterococcus*^*c*^
	Enterococcus faecalis
	Enterococcus faecium
	*Lactobacillus*^*d*^
	*Listeria*^*c*^
	Listeria monocytogenes
	*Micrococcus*
	*Cutibacterium* (*Propionibacterium*) *acnes*
	*Staphylococcus*^*c*^
	Staphylococcus aureus
	Staphylococcus epidermidis
	Staphylococcus lugdunensis
	*Streptococcus*^*c*^
	Streptococcus agalactiae
	Streptococcus anginosus group^*e*^
	Streptococcus pneumoniae
Antimicrobial resistance gene^*f*^	*mecA* (methicillin resistance)
	*mecC* (methicillin resistance)
	*vanA* (vancomycin resistance)
	*vanB* (vancomycin resistance)
Pan target	Pan Gram-Negative^*g*^
	Pan *Candida*^*h*^

a Bacillus cereus and Bacillus thuringiensis.

b Bacillus subtilis, Bacillus amyloliquefaciens, Bacillus atrophaeus, and Bacillus licheniformis.

c In the event that there is a species of *Enterococcus*, *Listeria*, *Staphylococcus*, or *Streptococcus* detected by the BCID-GP Panel, the genus-level target will always be detected along with the species that is identified (i.e., Staphylococcus epidermidis will appear on the ePlex BCID-GP Panel report as *Staphylococcus* spp. and Staphylococcus epidermidis).

d Lactobacillus casei, Lactobacillus paracasei, Lactobacillus rhamnosus, and Lactobacillus zeae.

e Streptococcus anginosus, Streptococcus intermedius, Streptococcus constellatus.

f Antimicrobial resistance genes will not be detected without a corresponding organism on the ePlex BCID-GP Panel. Antimicrobial resistance genes *mecA* and *mecC* will populate only in the event that a *Staphylococcus* sp. is detected. Likewise, *vanA* and *vanB* will populate only in the event that an *Enterococcus* sp. is detected.

g Detects up to 95% of Gram-negative bacteria, including (but not limited to) *Enterobacterales*, Acinetobacter, Ps*eudomonas*, *Bacteroides*, Stenotrophomonas maltophilia, *Serratia*, and *Neisseria*.

h Candida albicans, Candida glabrata, Candida parapsilosis, and Candida krusei.

### Comparator methods.

The comparator methods for organism identification consisted of standard laboratory procedures, including traditional culture, FDA-cleared MALDI-TOF MS (i.e., bioMérieux Vitek MS or Bruker Biotyper), and automated microbiological and biochemical techniques (e.g., Becton, Dickinson [BD] Phoenix, bioMérieux Vitek 2, or Siemens MicroScan). The majority (73.4%) of prospective fresh samples had organisms identified by an FDA-cleared MALDI-TOF MS system (Vitek MS or Bruker). The versions used by the sites are as follows: Johns Hopkins, the Bruker Microflex LT/SH IVD version 2.0; Detroit Medical Center, the Bruker Microflex LT/SH IVD version 2.0; Medical University of South Carolina (MUSC), Bruker Microflex LT/SH IVD version 4.0.11.0; Indiana University, Bruker Microflex LT/SH IVD version 3.2.14; Henry Ford Health System, Vitek MS version 2.2; and Tricore Laboratories, Bruker Microflex LT/SH IVD version 3.2.12.2. Prospective frozen samples were comprised of organisms identified by the bioMérieux Vitek 2 microbiology system (25.8%), Siemens MicroScan (23.3%), BD Phoenix (2.5%), or a wide variety of conventional manual methods. Organisms in the retrospective Gram-positive samples were identified primarily by the BD Phoenix (44.2%) or bioMérieux Vitek 2 (17.4%) microbiology system. Methods differed between the fresh and frozen samples due to sites changing methods between the collection time periods.

Due to known issues with organism identification, samples with *Corynebacterium*, Staphylococcus epidermidis, Staphylococcus hominis, or C. parapsilosis identified by standard laboratory procedures were confirmed using analytically validated PCR amplification assays followed by bidirectional sequencing (PCR/sequencing) or 16S rRNA gene sequencing (21–24).

The comparator methods for ARGs were analytically validated real-time PCR (qPCR) amplification assay(s) followed by bidirectional sequencing for samples with the associated organisms identified (i.e., *Staphylococcus* and *Enterococcus*).

### Method for resolution of discordant results.

Results from collected samples that were discordant between the BCID-GP Panel and the comparator method(s) (i.e., false negative or false positive) were tested with PCR/sequencing and/or 16S sequencing to determine the presence or absence of the organism. For organisms and ARGs included on the BCID-GP Panel, a false-negative result occurred when the comparator method identified an organism or ARG that was not detected with the BCID-GP Panel. A false-positive result occurred when the BCID-GP Panel detected an organism or ARG that the comparator method did not identify.

### Statistical methods.

Positive percent agreement (PPA) and negative percent agreement (NPA) with comparator method results were determined for each target detected by the BCID-GP Panel. PPA was calculated as 100 × number of TP/(number of TP + number of FN), and NPA was calculated as 100 × number of TN/(number of TN + number of FP), where TP is true positives, FN is false negatives, TN is true negatives, and FP is false positives. The two-sided 95% score confidence interval (CI) was calculated for PPA and NPA.

## RESULTS

### Demographic/sample information, sample disposition, and run/sample accountability.

Demographic information for the evaluable prospectively and retrospectively collected subject samples is provided in Table S2. Slightly more than 50% of the subjects were male, with a median age of 60 years.

Thirteen different blood culture bottle types from three manufacturers were used. Of the 10 clinical sites collecting patient samples, 6 used BD Bactec, 2 used Thermo Fisher Scientific VersaTREK, and 2 sites used both BD and bioMérieux BacT/Alert. There were no discernible trends for a specific bottle type (data not shown). Ninety percent of samples were either tested or frozen within 12 h of blood culture bottle positivity. The prevalence of BCID-GP Panel targets by age group during prospective collection is provided in Table S3.

A total of 719 prospectively collected, 1,070 retrospectively collected (includes both the Gram-positive samples and the non-Gram-positive samples) and 566 contrived samples were tested with the BCID-GP Panel, including 10 withdrawn samples (7 prospective and 3 retrospective) tested prior to withdrawal. Of the 2,354 samples initially tested with the BCID-GP Panel, 108 yielded invalid results, for an initial validity rate of 95.4%. After repeat testing, only 2 samples (1 contrived and 1 retrospective) of the 108 repeated as invalid, for a final validity rate of 99.9%. There were 1,297 evaluable clinical samples (including prospective and retrospective samples) and 565 contrived samples for the Gram-positive targets, as well as 1,777 evaluable clinical samples for the Pan targets.

### Panel performance.

There were 1,297 positive blood culture samples with Gram stains that displayed Gram-positive organisms, including 1,220 with only Gram-positive, 64 with mixed Gram-positive and Gram-negative organisms, 3 with mixed Gram-positive organisms and yeast, 3 with mixed Gram-positive organisms, Gram-negative organisms, and yeast, and 7 with Gram-variable organisms. The BCID-GP Panel correctly identified the organisms/ARGs identified by comparator methods in 93% of these samples. The overall sample accuracy where the BCID-GP Panel and comparator methods detected the same organisms/ARGs was 89% before resolution of discordant results. After correction for organisms/ARGs missed by comparator methods but detected by the BCID-GP Panel and confirmed by other methods, the overall sample accuracy was 91%. The overall weighted PPA across all 26 targets was 96%. These results exclude the 28 Gram-positive (2.2%) organisms representing 12 genera identified by comparator methods but not targeted by the BCID-GP Panel (Table S4). These 28 isolates included 6 *Clostridium* spp., 6 *Rothia* spp., 3 *Aerococcus* isolates, 2 *Microbacterium* isolates, 2 *Peptostreptococcus* spp., and single isolates representing 7 different taxa and 2 organisms not identified beyond Gram stain morphology.

Positive percent agreement and NPA with 95% CI of the BCID-GP Panel targets with comparator methods are provided in Table 2 for Gram-positive targets. Table S5 details the performance of the BCID-GP Panel for all samples stratified by the species detected by the comparator methods or in the known contrived samples. Table S6 summarizes the BCID-GP Panel clinical performance after resolution of discordant results. Specific details of the discordant results are provided throughout the narrative summaries below.

**TABLE 2:** BCID-GP Panel bacterial pathogen clinical performance

Target	Clinical samples^*a*^				Contrived samples				Combined samples			
	Sensitivity/PPA		Specificity/NPA		Sensitivity/PPA		Specificity/NPA		Sensitivity/PPA		Specificity/NPA	
	No. of TP/no. of TP + FN	% (95% CI)	No. of TN/no. of TN + FP	% (95% CI)	No. of TP/no. of TP + FN	% (95% CI)	No. of TN/no. of TN + FP	% (95% CI)	No. of TP/no. of TP + FN	% (95% CI)	No. of TN/no. of TN + FP	% (95% CI)
B. cereus group	11/12	91.7 (64.6–98.5)	1,285/1,285	100 (99.7–100)	46/46	100 (92.3–100)	519/519	100 (99.3–100)	57/58	98.3 (90.9–99.7)	1,804/1,804	100 (99.8–100)
												
*Enterococcus*	200/208	96.2 (92.6–98.0)	1,088/1,089	99.9 (99.5–100)	126/126	100 (97.0–100)	439/439	100 (99.1–100)	326/334	97.6 (95.3–98.8)	1,527/1,528	99.9 (99.6–100)
E. faecalis	131/139	94.2 (89.1–97.1)	1,157/1,157	100 (99.7–100)	52/52	100 (93.1–100)	513/513	100 (99.3–100)	183/191	95.8 (92.0–97.9)	1,670/1,670	100 (99.8–100)
E. faecium	63/65	96.9 (89.5–99.2)	1,223/1,231	99.4 (98.7–99.7)	60/60	100 (94.0–100)	505/505	100 (99.2–100)	123/125	98.4 (94.4–99.6)	1,728/1,736	99.5 (99.1–99.8)
	
*Listeria*	2/2	100 (34.2–100)	1,294/1,295	99.9 (99.6–100)	74/75	98.7 (92.8–99.8)	490/490	100 (99.2–100)	76/77	98.7 (93.0–99.8)	1,784/1,785	99.9 (99.7–100)
L. monocytogenes	2/2	100 (34.2–100)	1,295/1,295	100 (99.7–100)	46/46	100 (92.3–100)	519/519	100 (99.3–100)	48/48	100 (92.6–100)	1,814/1,814	100 (99.8–100)
												
*Staphylococcus*	632/647	97.7 (96.2–98.6)	640/650	98.5 (97.2–99.2)	105/105	100 (96.5–100)	460/460	100 (99.2–100)	737/752	98.0 (96.7–98.8)	1,100/1,110	99.1 (98.3–99.5)
S. aureus	282/291	96.9 (94.2–98.4)	920/926	99.4 (98.6–99.7)	59/59	100 (93.9–100)	506/506	100 (99.2–100)	341/350	97.4 (95.2–98.6)	1,426/1,432	99.6 (99.1–99.8)
S. epidermidis	148/159	93.1 (88.0–96.1)	1,020/1,039	98.2 (97.2–98.8)	1/1	100 (20.7–100)	564/564	100 (99.3–100)	149/160	93.1 (88.1–96.1)	1,584/1,603	98.8 (98.2–99.2)
*S. lugdunensis*	6/6	100 (61.0–100)	1,190/1,192	99.8 (99.4–100)	45/45	100 (92.1–100)	519/520	99.8 (98.9–100)	51/51	100 (93.0–100)	1,709/1,712	99.8 (99.5–99.9)
	
*Streptococcus*	274/283	96.8 (94.1–98.3)	1,005/1,014	99.1 (98.3–99.5)	57/57	100 (93.7–100)	508/508	100 (99.2–100)	331/340	97.4 (95.0–98.6)	1,513/1,522	99.4 (98.9–99.7)
S. agalactiae	46/48	95.8 (86.0–98.8)	1,220/1222	99.8 (99.4–100)	8/8	100 (67.6–100)	557/557	100 (99.3–100)	54/56	96.4 (87.9–99.0)	1,777/1,779	99.9 (99.6–100)
*S. anginosus* group	42/45	93.3 (82.1–97.7)	1,222/1,225	99.8 (99.3–99.9)	23/23	100 (85.7–100)	542/542	100 (99.3–100)	65/68	95.6 (87.8–98.5)	1,764/1,767	99.8 (99.5–99.9)
S. pneumoniae	66/69	95.7 (88.0–98.5)	1,198/1,200	99.8 (99.4–100)	0/0		565/565	100 (99.3–100)	66/69	95.7 (88.0–98.5)	1,763/1,765	99.9 (99.6–100)
S. pyogenes	27/28	96.4 (82.3–99.4)	1,241/1,241	100 (99.7–100)	26/26	100 (87.1–100)	539/539	100 (99.3–100)	53/54	98.1 (90.2–99.7)	1,780/1,780	100 (99.8–100)

a Clinical samples include prospective and retrospective samples.

In the clinical arms of the study, only 12 Bacillus cereus group organisms were recovered by SOC methods (Table 2). For those 12 samples, 11 were correctly identified by the BCID-GP Panel (91.7% PPA) and the NPA was 100%. Overall results with the contrived samples included a PPA of 98.3% and NPA of 100%.

A total of 334 enterococci, including 191 Enterococcus faecalis samples and 125 Enterococcus faecium samples, were tested with an overall PPA of 97.6% and NPA of 99.9%. E. faecalis was more frequently isolated from clinical samples (*n* = 139) than E. faecium (*n* = 65). *Enterococcus* was detected in one false-positive prospective sample using PCR/sequencing. When considering the major species separately, the overall PPA for E. faecalis was 95.8% and the NPA was 100% (Table 2). E. faecalis was not detected in four of the eight false-negative clinical samples using PCR/sequencing. Three contained E. faecium, while Lactococcus lactis was isolated from one sample. For E. faecium, the overall PPA was 98.4% and the NPA was 99.5% (Table 2). Five of the eight false-positive samples were detected by PCR/sequencing as E. faecium.

Only two *Listeria* clinical samples were identified by SOC methods, so 75 contrived samples containing several *Listeria* species (i.e., Listeria innocua, L. ivanovii, L. monocytogenes, L. seeligeri, and L. welshimeri) were included to supplement the data. The overall PPA and NPA for the *Listeria* target were 98.7% and 99.9%, respectively. The two clinical samples grew L. monocytogenes; therefore, 46 of 48 of the samples for this organism were contrived. For the L. monocytogenes target, the two clinical samples were detected by the BCID-GP Panel and the assay accurately detected all contrived samples as noted in Table 2.

As expected, *Staphylococcus* targets were abundant during the clinical arms of the study, with a total of 647 staphylococci detected by SOC testing. Eighteen species or subspecies of staphylococci were included (Table S5). The BCID-GP Panel detected 632 of the staphylococci, with a PPA for the clinical samples of 97.7%; the NPA was 98.5% (Table 2). *Staphylococcus* was not detected in five false-negative samples using PCR/sequencing. These samples contained a variety of Gram-negative and Gram-positive organisms (one contained Escherichia coli, one Klebsiella pneumoniae, and one S. salivarius, and two had no other organisms detected). For the 10 false-positive samples, *Staphylococcus* species were detected in 9 by PCR/sequencing. All 105 contrived samples (59 S. aureus, 1 S. epidermidis, and 45 *S. lugdunensis*) were accurately identified by the BCID-GP Panel.

There were 291 total S. aureus isolates recovered by SOC methods, with 98.8% overall accuracy by the BCID-GP Panel. The PPA was 96.9% for all clinical samples (prospective and retrospective), and the NPA was 99.4% (Table 2). There were nine false-negative samples compared to SOC methods. S. aureus was not detected by PCR/sequencing in three false-negative prospective samples, but Staphylococcus simulans, Streptococcus agalactiae, and Klebsiella pneumoniae were detected. However, for five of the six false-positive samples, S. aureus was detected using PCR/sequencing. All 59 contrived samples were accurately identified by the BCID-GP Panel, for a PPA and NPA of 100% (Table 2).

The data for S. epidermidis were less robust than the S. aureus data but still in the acceptable range (PPA > 90%). A total of 159 S. epidermidis isolates were detected by SOC methods. The overall PPA and NPA (including the singular contrived sample) were 93.1% and 98.8%, respectively. There were six false-negative samples that contained a variety of different staphylococcal species as per testing by PCR/sequencing, including four samples identified as S. aureus (originally misidentified as S. epidermidis by the SOC method). The other staphylococci identified were Staphylococcus capitis and Staphylococcus pettenkoferi.

Standard-of-care methods detected six *S. lugdunensis*-positive blood cultures, and all six were correctly identified by the BCID-GP Panel. All 45 contrived samples were also accurately detected. There were two false positives among the clinical samples which, when analyzed by PCR/sequencing, were found to have *S. lugdunensis* present. Overall, the PPA was 100% and the NPA was 99.8% (Table 2).

The BCID-GP Panel detects the *Streptococcus* genus, with the ability to distinguish four species or groups—S. agalactiae, S. anginosus group, S. pneumoniae, and S. pyogenes. Sixteen species or groups within the *Streptococcus* genus were detected during the study (Table S5). The PPA ranged from 95.6% for the *S. anginosus* group to 98.1% for S. pyogenes. Similar to the data for the other Gram-positive targets, the NPAs were high, ranging from 99.4% to 100% (Table 2). There were 13 samples with 16 false-positive results across the five *Streptococcus* targets, whereby the respective organism was detected by PCR/sequencing in 11 (8 *Streptococcus* species, 1 S. agalactiae, 1 Streptococcus intermedius, and 1 S. pneumoniae). Of the 16 samples that yielded 18 false-negative results, 4 samples had no streptococci detected. Streptococcus mitis was detected by PCR/sequencing in one false-negative S. agalactiae sample, one isolate each of Granulicatella adiacens, S. dysgalactiae, and S. lutetiensis was detected in three false-negative *S. anginosus* group samples, and of three false negatives for S. pneumoniae, two were positive for S. mitis and one was positive for *S. anginosus* by PCR/sequencing.

The data for the Pan targets are presented in Table 3. No contrived samples with Gram-negative or fungal organisms were tested. One of the four *Candida* species contained in the Pan *Candida* portion of the assay was detected by SOC methods in a total of 105 clinical samples. The overall PPA was 92.4%, and the NPA was 99.9% (Table 4). Recall that clinical samples without positive Gram stain results were included to increase the sample size to evaluate the Pan targets. Of the eight false-negative samples, only two had positive Gram stain results intended to be tested with the BCID-GP Panel. Notably, the Gram stain only indicated Gram-positive organisms and missed the presence of yeasts that were later identified, with one sample having C. glabrata verified by PCR/sequencing. The remaining six false-negative samples had fungal and/or negative Gram stain results, of which four had C. albicans verified by PCR/sequencing. C. glabrata was detected in one of two false-positive samples using PCR/sequencing. A Gram-negative organism was identified in 441 clinical samples, and the Pan Gram-Negative target demonstrated an overall PPA of 95.7% and NPA of 99.6%. Of the 19 false-negative samples, only 8 had positive Gram stain results intended to be tested with the BCID-GP Panel, and two had Gram-negative organisms verified by PCR/sequencing. The remaining 11 false-negative samples had negative Gram stain results, of which 9 had Gram-negative organisms verified by PCR/sequencing. By the comparator methods, the organisms that were not detected included the following: one Achromobacter xylosoxidans subsp. *xylosoxidans* isolate, two Acinetobacter baumannii isolates, two Bacteroides fragilis isolates, four Bacteroides thetaiotaomicron isolates, one Campylobacter gracilis isolate, one Delftia acidovorans isolate, two K. pneumoniae isolates, one Ochrobactrum anthropi isolate, one Proteus mirabilis isolate, one Providencia stuartii isolate, one Pseudomonas aeruginosa isolate, and one isolate each of *Wolinella* and *Veillonella*. Of note, the assay correctly identified three samples containing Salmonella enterica subsp. *enterica* serovar Typhi. Of seven false-positive samples, two samples had E. coli and K. pneumoniae, respectively, detected, whereas in the other five samples no Gram-negative organisms were detected using PCR/sequencing (Table 3). Of the 1,220 samples with positive Gram stain results, 11 (0.9%) were coinfected with a Gram-negative bacterium or *Candida* detected by either the BCID-GP Panel (*n* = 6), SOC (*n* = 3), or both (*n* = 2). The BCID-GP Panel alone detected five Gram-negative organisms and one *Candida* organism, with PCR/sequencing confirming one to be K. pneumoniae. Both methods detected Gram-negative E. coli in two samples. In 3 samples, SOC alone detected *Veillonella*, C. glabrata, and C. parapsilosis.

**TABLE 3:** Clinical performance of the BCID-GP Panel Pan targets with comparator methods

Target	Clinical samples^*a*^				Contrived samples				Combined samples			
	Sensitivity/PPA		Specificity/NPA		Sensitivity/PPA		Specificity/NPA		Sensitivity/PPA		Specificity/NPA	
	No. of TP/no. of TP + FN	% (95% CI)	No. of TN/no. of TN + FP	% (95% CI)	No. of TP/no. of TP + FN	% (95% CI)	No. of TN/no. of TN + FP	% (95% CI)	No. of TP/no. of TP + FN	% (95% CI)	No. of TN/no. of TN + FP	% (95% CI)
Pan *Candida*	97/105	92.4 (85.7–96.1)	1,670/1,672	99.9 (99.6–100)	0/0		565/565	100 (99.3–100)	97/105	92.4 (85.7–96.1)	2,235/2,237	99.9 (99.7–100)
Pan Gram-Negative	422/441	95.7 (93.4–97.2)	1,329/1,336	99.5 (98.9–99.7)	0/0		565/565	100 (99.3–100)	422/441	95.7 (93.4–97.2)	1,894/1,901	99.6 (99.2–99.8)

a Clinical samples include prospective and retrospective samples.

**TABLE 4:** Detection of antimicrobial resistance gene targets by the BCID-GP Panel versus comparator methods

Target^*b*^	Clinical samples^*a*^				Contrived samples				Combined samples			
	Sensitivity/PPA		Specificity/NPA		Sensitivity/PPA		Specificity/NPA		Sensitivity/PPA		Specificity/NPA	
	No. of TP/no. of TP + FN	% (95% CI)	No. of TN/no. of TN + FP	% (95% CI)	No. of TP/no. of TP + FN	% (95% CI)	No. of TN/no. of TN + FP	% (95% CI)	No. of TP/no. of TP + FN	% (95% CI)	No. of TN/no. of TN + FP	% (95% CI)
*mecA*	401/413	97.1 (95.0–98.3)	223/234	95.3 (91.8–97.4)	11/11	100 (74.1–100)	94/94	100 (96.1–100)	412/424	97.2 (95.1–98.4)	317/328	96.6 (94.1–98.1)
*mecC*	0/0		647/647	100 (99.4–100)	49/49	100 (92.7–100)	56/56	100 (93.6–100)	49/49	100 (92.7–100)	703/703	100 (99.5–100)
*vanA*	61/65	93.8 (85.2–97.6)	141/143	98.6 (95.0–99.6)	60/60	100 (94.0–100)	66/66	100 (94.5–100)	121/125	96.8 (92.1–98.7)	207/209	99.0 (96.6–99.7)
*vanB*	1/1	100 (20.7–100)	207/207	100 (98.2–100)	52/52	100 (93.1–100)	74/74	100 (95.1–100)	53/53	100 (93.2–100)	281/281	100 (98.7–100)

a Clinical samples include prospective and retrospective samples.

b Only performance for *Staphylococcus* spp. (*mecA* and *mecC*) and *Enterococcus* spp. (*vanA* and *vanB*) was assessed for these antimicrobial resistance genes.

Table 4 contains the data for the antimicrobial resistance gene targets. Similar to the case with other commercially available highly multiplexed blood culture identification tests, if there are multiple Gram-positive cocci in a sample that is *mecA* positive, there is no way to link the gene to the detected species if they are all staphylococci (19, 25, 26). There were 413 *mecA*-positive staphylococci detected in the clinical study by the comparator methods, and 11 contrived specimens were also tested. The overall PPA and NPA were 97.2% and 96.6%, respectively. Among the clinical specimens, there were 12 false-negative samples. However, additional testing indicated that two of the false-negative samples were contaminated with *mecA* during the original extraction process for the comparator method testing. Samples were reextracted twice and upon repeat analysis, *mecA* was not present. Eleven false-positive *mecA* samples were also noted, and in four of these (three prospective and one retrospective), *mecA* was found to be present using an FDA-cleared multiplex PCR assay. Among the clinical samples, 194 Staphylococcus aureus isolates were *mecA* positive by comparator methods, with 190 *mecA* genes detected by the BCID-GP Panel (PPA, 97.9%). Up to two coagulase-negative *Staphylococcus* species (CoNS) were detected in 154 samples that were *mecA* positive by comparator methods (BCID-GP Panel detected *mecA* in 149; PPA, 96.8%). These included S. epidermidis (*n* = 98), *S. capitis* (*n* = 7), S. haemolyticus (*n* = 5), S. hominis (*n* = 27), S. auricularis (*n* = 1), S. saprophyticus (*n* = 1), *S. simulans* (*n* = 1), S. epidermidis/S. hominis (*n* = 9), and one isolate each of S. epidermidis/*S. capitis*, S. epidermidis/S. lugdunensis, *S. capitis*/S. hominis, S. cohnii/S. hominis, and *S. haemolyticus*/S. hominis (data not shown).

This is the first commercial panel to detect *mecC* among the staphylococci. Interestingly, there were no *mecC*-containing staphylococci detected during the clinical study, confirming reports of low prevalence of *mecC* in the United States (27, 28). The assay detected *mecC* in all of the 49 contrived samples tested (Table 4).

The major vancomycin resistance determinant among the clinical enterococci was *vanA* (*n* = 65). In terms of species distribution for *vanA*-positive samples, E. faecium accounted for 75% and E. faecalis for the majority of the others. Among the 65 enterococcal samples for which comparator methods detected *vanA*, 13 had E. faecalis, 49 had E. faecium, 2 samples had dual E. faecalis/E. faecium, and 1 contained Enterococcus avium. The BCID-GP Panel detected *vanA* in 61/65 samples (PPA, 93.8%). There were three E. faecalis isolates and one *E. avium* isolate that were determined to be *vanA* positive by comparator methods but were missed by the BCID-GP Panel. The clinical data were supplemented with 60 contrived samples (Table S1). Overall, the BCID-GP Panel identified 121 of the 125 (96.8%) *vanA*-positive samples. The overall NPA was 99.0%. For two of the false-negative samples, a *vanA* signal was present, but without the presence of an associated *Enterococcus* organism detected by the BCID-GP Panel, *vanA* was not reported. Similarly, additional testing indicated that two of the false-negative samples were contaminated with *vanA* during the original extraction process for the comparator method testing. Samples were reextracted twice and *vanA* was not present. When tested using the FDA-cleared multiplex PCR assay mentioned above, one of these samples did not have *vanA* detected. *vanA* was also detected in one of the two false-positive samples that were tested with the same FDA-cleared multiplex PCR assay (Table 4). There was only one *vanB*-positive sample containing E. faecalis among the clinical samples tested; the assay accurately detected *vanB* in that sample and all 52 contrived samples.

### Contamination rule-out targets.

The BCID-GP Panel includes five targets, Bacillus subtilis group, *Corynebacterium*, *C. acnes*, *Lactobacillus*, and *Micrococcus*, where assay design favored very high specificity over sensitivity because the primary clinical utility of these targets is to rule out contamination (Table 5). For these five targets, the NPA in clinical samples and overall in all samples was at least 99.8%. For the *Corynebacterium* target, comparator methods identified 14 different species (Table S5). Of 11 samples with false-negative results, 7 were not identified to the species level by the laboratories performing the assay, resulting in an overall PPA of 84.5%. The other four species that were not detected by the BCID-GP Panel were C. afermentans, C. jeikeium, C. pseudotuberculosis, and C. urealyticum.

**TABLE 5:** Contamination rule-out targets

Target	Clinical samples^*a*^				Contrived samples				Combined samples			
	Sensitivity/PPA		Specificity/NPA		Sensitivity/PPA		Specificity/NPA		Sensitivity/PPA		Specificity/NPA	
	No. of TP/no. of TP + FN	% (95% CI)	No. of TN/no. of TN + FP	% (95% CI)	No. of TP/no. of TP + FN	% (95% CI)	No. of TN/no. of TN + FP	% (95% CI)	No. of TP/no. of TP + FN	% (95% CI)	No. of TN/no. of TN + FP	% (95% CI)
B. subtilis group	2/2	100 (34.2–100)	1,294/1,294	100 (99.7–100)	50/50	100 (92.9–100)	515/515	100 (99.3–100)	52/52	100 (93.1–100)	1,809/1,809	100 (99.8–100)
*Corynebacterium*	40/51	78.4 (65.4–87.5)	1,244/1,246	99.8 (99.4–100)	20/20	100 (83.9–100)	545/545	100 (99.3–100)	60/71	84.5 (74.3–91.1)	1,789/1,791	99.9 (99.6–100)
Cutibacterium acnes	18/20	90.0 (69.9–97.2)	1,275/1,277	99.8 (99.4–100)	25/26	96.2 (81.1–99.3)	539/539	100 (99.3–100)	43/46	93.5 (82.5–97.8)	1,814/1,816	99.9 (99.6–100)
*Lactobacillus*	13/13	100 (77.2–100)	1,282/1,284	99.8 (99.4–100)	32/33	97.0 (84.7–99.5)	532/532	100 (99.3–100)	45/46	97.8 (88.7–99.6)	1,814/1,816	99.9 (99.6–100)
*Micrococcus*	39/44	88.6 (76.0–95.0)	1,252/1,253	99.9 (99.5–100)	27/27	100 (87.5–100)	538/538	100 (99.3–100)	66/71	93.0 (84.6–97.0)	1,790/1,791	99.9 (99.7–100)

a Clinical samples include prospective and retrospective samples.

An investigation into these 11 false-negative results was performed using PCR/sequencing and 16S rRNA sequencing. For five samples, PCR/sequencing was negative and 16S rRNA sequencing detected *C. acnes*, Lactobacillus fermentum, Macrococcus caseolyticus, and *S. pettenkoferi* (the fifth sample was not tested with 16S rRNA sequencing). Five samples had *Corynebacterium* detected by PCR/sequencing and/or 16S rRNA sequencing. The remaining sample had indeterminate results. *Corynebacterium* was detected in two false-positive clinical samples using PCR/sequencing.

For *C. acnes*, the overall PPA was 93.5%. PCR/sequencing detected *C. acnes* in one false-positive clinical sample. In the two false-negative samples, PCR/sequencing detected *C. acnes*.

*Lactobacillus* was detected in all of the positive clinical samples, and there was only one false-negative contrived sample, for an overall PPA of 97.8% (45/46). There were two false-positive samples, and PCR/sequencing detected Lactobacillus casei in one sample.

Forty-four clinical samples contained *Micrococcus* species; the assay failed to detect five of them, four of which were identified to the genus level only by the comparator methods, and the fifth one was identified as Micrococcus luteus/Micrococcus lylae (data not shown). *Micrococcus* was not detected in three false-negative clinical samples using PCR/sequencing; one Brevibacterium ravenspurgense isolate, one Nesterenkonia halotolerans isolate, and one *S. pettenkoferi* isolate were detected. Consistent with the performance with the other contamination targets, the NPA overall was close to 100%.

Table S7 summarizes single infections with discordant results, that is, BCID-GP Panel false-negative or false-positive results compared to the comparator methods. The results for mixed infections are described in detail below, and concordant mixed infections are highlighted in Table 6; discordant mixed infections are catalogued in Table S8.

**TABLE 6:** Coinfections detected by comparator methods that were concordant with the BCID-GP Panel targets

Organism				ARG(s)	No. of samples
1	2	3	4		
*A. baumannii*^*a*^	E. faecium			*vanA*	1
*A. baumannii*^*a*^	E. faecium	*Staphylococcus*		*mecA*, *vanA*	1
*A. baumannii*^*a*^	S. aureus				1
*A. baumannii*^*a*^	*Staphylococcus*			*mecA*	1
Aerococcus sanguinicola^*b*^	*Corynebacterium*	Staphylococcus saprophyticus			1
Aerococcus viridans^*b*^	Staphylococcus hominis				1
B. fragilis^*a*^	*Clostridium* spp.^*b*^				1
B. fragilis^*a*^	*S. anginosus* group				1
C. albicans^*a*^	S. epidermidis			*mecA*	1
C. glabrata^*a*^	S. pneumoniae				1
C. krusei^*a*^	S. epidermidis			*mecA*	1
C. parapsilosis^*a*^	E. faecalis				1
Citrobacter braakii^*a*^	Streptococcus oralis				1
Citrobacter koseri^*a*^	E. faecalis				1
*Corynebacterium*	S. epidermidis			*mecA*	1
*Corynebacterium*	*Staphylococcus*			*mecA*	1
E. cloacae^*a*^	E. faecalis				1
*E. cloacae*^*a*^	E. faecium			*vanA*	1
*E. cloacae*^*a*^	E. faecium	Staphylococcus hominis		*mecA*, *vanA*	1
*E. cloacae*^*a*^	*S. anginosus* group				1
E. coli^*a*^	E. faecalis				3
E. coli^*a*^	E. faecalis	K. pneumoniae^*a*^			1
E. coli^*a*^	E. faecalis	P. mirabilis^*a*^			2
E. coli^*a*^	E. faecium				2
E. coli^*a*^	K. oxytoca^*a*^	Streptococcus infantarius			1
E. coli^*a*^	Lactococcus lactis^*b*^				1
E. coli^*a*^	P. mirabilis^*a*^	Providencia stuartii^*a*^	*S. anginosus* group		1
E. coli^*a*^	S. agalactiae				1
E. coli^*a*^	*S. anginosus* group				1
E. coli^*a*^	S. pneumoniae				1
E. coli^*a*^	Streptococcus bovis				1
E. faecalis	E. faecium				2
E. faecalis	E. faecium			*vanA*	2
E. faecalis	K. pneumoniae^*a*^				2
E. faecalis	M. morganii^*a*^			*vanA*	1
E. faecalis	*P. mirabilis*^*a*^				1
E. faecalis	S. aureus			*mecA*	1
E. faecalis	S. epidermidis			*mecA*	1
E. faecalis	*S. maltophilia*^*a*^			*vanA*	1
E. faecalis	S. marcescens^*a*^				1
E. faecalis	*Staphylococcus* (CoNS)			*mecA*	1
E. faecium	K. pneumoniae^*a*^				1
E. faecium	Moraxella catarrhalis^*a*^	Pediococcus pentosaceus^*b*^		*vanA*	1
E. faecium	*P. mirabilis*^*a*^			*vanA*	1
E. faecium	*Pseudomonas*^*a*^			*vanA*	1
E. faecium	S. aureus			*mecA*, *vanA*	1
E. faecium	S. epidermidis	Staphylococcus haemolyticus		*mecA*, *vanA*	1
Enterobacter aerogenes^*a*^	*S. anginosus* group				1
Enterococcus avium	*S. anginosus* group				1
*K. oxytoca*^*a*^	*S. anginosus* group				1
K. pneumoniae^*a*^	S. aureus				1
K. pneumoniae^*a*^	Staphylococcus haemolyticus	Nonfermenting Gram-negative bacilli^*a*^			1
Lactobacillus rhamnosus	Pediococcus acidilactici^*b*^				1
*Micrococcus*	*Pseudoclavibacter*^*b*^				1
*Moraxella catarrhalis*^*a*^	S. pneumoniae				1
*P. aeruginosa*^*a*^	*P. mirabilis*^*a*^	*Streptococcus*, viridans group			1
*Peptostreptococcus* species^*b*^	*Staphylococcus*				1
Rothia mucilaginosa^*b*^	S. epidermidis				1
S. agalactiae	S. aureus				2
S. agalactiae	S. aureus			*mecA*	1
S. agalactiae	S. aureus	*Streptococcus*, viridans group			1
S. aureus	S. epidermidis			*mecA*	1
S. aureus	Staphylococcus capitis				1
S. aureus	Streptococcus mitis			*mecA*	1
S. epidermidis	Staphylococcus capitis			*mecA*	1
S. epidermidis	Staphylococcus hominis				3
S. epidermidis	Staphylococcus hominis			*mecA*	6
S. epidermidis	Staphylococcus hominis	Staphylococcus warneri			1
S. epidermidis	Staphylococcus hominis	Streptococcus parasanguinis		*mecA*	1
S. epidermidis	Streptococcus parasanguinis				1

a The BCID-GP Panel detected this organism with a Pan target.

b Off-panel organism not targeted by the BCID-GP Panel.

### Mixed infections.

Overall, 184 mixed infections were identified by comparator methods and/or the BCID-GP Panel. Codetections of on-panel targets identified by comparator methods that were concordant with the BCID-GP Panel in clinical samples are provided in Table 6. The BCID-GP Panel correctly identified on-panel targets in 85 (46%) samples. Of these, 70 (82%) had two organisms, 14 (16%) had three organisms, and 1 (%) had four organisms. Of the 184 codetected samples, 99 (54%) discordant samples are provided in Table S8 and consist of codetections that included an organism/ARG not detected by the BCID-GP Panel (i.e., false negative) in 58 samples or an organism/ARG that was detected by the BCID-GP Panel but not identified by comparator methods (i.e., false positive) in 65 samples (some samples had both false-negative and false-positive detections). Thirty-one (48%) of the 65 false-positive detections were confirmed and 23 (40%) of the 58 false-negative detections were not confirmed by methods for resolving discordant results.

## DISCUSSION

Early pathogen identification and determination of resistance are essential to successful treatment outcome in patients with bacteremia and sepsis (29–31). Although direct detection of pathogens from whole blood has been realized on a limited basis in the United States and Europe, because of many challenges with their performance and interpretation, and the costs associated with implementation, blood cultures remain the major diagnostic method for detection of bloodstream infections (31, 32).

Several pathogen-specific and broad-based syndromic panels exist for rapid identification and for rapid resistance marker detection from positive blood culture bottles (12, 18, 19, 33). The GenMark Dx ePlex BCID-GP Panel offers the broadest panel to date for the identification of Gram-positive organisms, as it targets 13 Gram-positive cocci, 7 Gram-positive rods, and 4 antimicrobial resistance genes. In this study, the GenMark Dx IUO BCID-GP Panel compared favorably to SOC and reference molecular testing, with an overall accuracy for on-panel organisms of 89% before resolution of discordant results. This is below the 96% overall sample agreement for a single-center clinical study on the ePlex BCID-GP research-use-only (RUO) Panel (34) performed in Belgium. However, this performance is very similar to the overall accuracies reported for the Verigene (Luminex) and FilmArray (BioFire) BCID panels (19, 35, 36). The ePlex system was also reliable, with an initial panel failure rate of 4.6% and only 2 samples repeating as invalid on repeat testing. There are no differences between the IUO BCID-GP Panel evaluated in this study and the now FDA-cleared ePlex BCID-GP Panel that is commercially available.

The panel is highly inclusive. Only 2.2% of clinical isolates identified by SOC methods were not targeted by the BCID-GP Panel (Table S4), in contrast to 7.5% to 11.9% reported for other currently available commercial assays (19, 25, 26). Most of these organisms are opportunistic pathogens that occasionally are associated with clinically significant bacteremia. Additionally, genus-level calls along with further identification to the species level within the genus may provide opportunity for more targeted therapeutic interventions depending on an institution’s antibiogram. For instance, the BCID-GP Panel can provide an *Enterococcus* genus result, which may prompt further investigation by the laboratory to rule out less common species of *Enterococcus*, such as *E. avium*. The BCID-GP Panel can likewise rule out E. faecium or E. faecalis in such cases or provide a specific identification in the event that one of the aforementioned species is present.

The BCID-GP Panel is unique in its inclusion of the Bacillus cereus group. This Gram-positive spore-forming rod is ubiquitous in the environment, and while it is best known for its association with foodborne infections, it can cause a variety of nongastrointestinal diseases, some of which are quite severe (37, 38). Bacteremia can lead to sepsis and associated high mortality (37, 38). B. cereus bloodstream infections are often nosocomial and the result of infected central venous catheters, as this organism can produce significant biofilms (37). Injection drug users are another population at risk for bacteremia and endocarditis (37, 38). Studies of immunocompromised hosts, especially those patients with hematological malignancies, have shown complicated outcomes, including metastatic neurological events such as meningoencephalitis and cerebral abscesses (37, 38). Given its propensity for severe infections in hospitalized patients and its antimicrobial resistance profile due to broad beta-lactamase production, more rapid identification of this organism may have significant impact on the time to appropriate therapy.

*Clostridium* species were among the most commonly missed Gram-positive organisms not targeted by this panel and are typically the second most common anaerobes isolated from blood cultures, behind the B. fragilis group (39, 40). Isolation of *Clostridium* species from blood cultures may represent contamination or transient or clinically insignificant bacteremia; however, it can also be an indicator of severe life-threatening infections requiring prompt identification and treatment (41, 42). For these reasons, consideration should be given to including a *Clostridium* target in future iterations of BCID panels (43).

Resistance gene detection and/or rapid phenotypic testing is important for early optimization of treatment. Several studies have demonstrated that when either of these is combined with stewardship interventions, the median time to optimal therapy, especially for methicillin-susceptible S. aureus (MSSA) and *Enterococcus* species, can be shortened by 14 h to more than 24 h (11, 13, 44, 45). In this study, *vanA* prevalence was low (*n* = 8 *vanA* enterococci) among the prospective samples, and no *vanB*-containing enterococci were detected. Distinguishing *vanA* from *vanB* is important in predicting susceptibility to glycopeptides. *vanA*-containing enterococci are resistant to both vancomycin and teicoplanin, whereas *vanB*-containing enterococci remain susceptible to teicoplanin (46). Performance among all samples, both clinical and contrived, revealed PPA and NPA for *vanA* of 96.8% and 99.0%, respectively, and 100% PPA and NPA for *vanB* detection, similar to the performance reported for other rapid blood culture panels and the only other publication on the ePlex BCID Panels (25, 26, 34). Likewise, the overall PPA and NPA for *mecA* detection of 97.1% and 95.3%, respectively, in the clinical samples among staphylococci are similar to those reported for other nucleic acid-based rapid methods (25, 26). This is the first commercially available rapid multiplex PCR panel able to identify *S. lugdunensis* along with the presence of a *mecA* gene. *S. lugdunensis* is an important species of staphylococci to identify due to its more aggressive nature than those of other coagulase-negative staphylococci (47). In addition, accurate identification is needed because the Clinical and Laboratory Standards Institute breakpoints for this organism follow the guidelines for S. aureus (47). Knowing whether *mecA* is present helps ensure earlier targeted therapy and hopefully a successful clinical outcome. The frequency of *mecA* is still very low in this species compared to those in other CoNS, but recently, two clones of *mecA*-positive *S. lugdunensis* (sequence type 38 [ST38] and ST44) were described that tested susceptible to cefoxitin and were shown by whole-genome sequencing to possess SCC*mec* type IVa(2b) with variations on the J3 region (47).

Also, unique to this panel is the detection of *mecC*, a *mec* variant first described in 2007 as a cause of bovine mastitis that has only 69% nucleotide sequence homology with *mecA* (48, 49). *mecC*-positive S. aureus appears to be increasing among isolates causing serious disease, including bacteremia, in humans and animals throughout Europe (48–53), although it was not detected among any S. aureus isolates in this study. *mecC*-producing S. aureus is not detected by the PBP-2a assays, as it encodes a different penicillin binding protein (48, 54). Also, at least one report demonstrated variable performance among the automated susceptibility testing platforms for its detection (55). At present, molecular methods appear to be the most reliable methods of *mecC* detection.

Several studies have assessed the clinical and economic impacts of BCID panels (33). Overall, these studies have shown a decrease in both the time to organism identification and the time to optimization of antimicrobial therapy. However, the results regarding impact of these panels on rates of mortality and hospital lengths of stay have been inconclusive. In a meta-analysis of 31 studies, the implementation of rapid blood culture diagnostics, including rapid molecular BCID panels, was associated with a lower mortality rate, a shorter time to effective therapy, and a decreased length of stay than with use of conventional microbiological methods in patients with bloodstream infections (15). Hospital-specific variables such as patient populations, local rates of antimicrobial resistance, and effectiveness of antimicrobial stewardship programs are likely to influence the clinical impact of rapid blood culture diagnostics. BCID panels and other rapid blood culture diagnostics have the greatest impact when the results are reported rapidly and appropriately acted on by clinicians with guidance from an antibiotic stewardship program (12, 56).

The GenMark Dx ePlex BCID-GP Panel is unique among the positive blood culture syndromic panels in targeting potential contaminants. Several studies report the impact of blood culture contamination on patient care and laboratory efficiencies (57–60). In a report from the College of American Pathologists in 2005, the overall mean blood culture contamination rate among 356 participating institutions was 2.89%, with a range of 2.15% to 3.67% (61). The estimated additional cost per patient was $5,506 (U.S. dollars). Other studies have determined that the total cost of extra hospital days in adult populations in the United States ranges from $1,372 to $2,200, resulting in significant additional expenditures—$1.8 million to $1.9 million—in medical costs per institution (61–63). Therefore, with the inclusion of the “contamination rule-outs” on this panel, rapid differentiation of contaminated blood cultures from true bacteremia is likely to have potential patient and laboratory benefits.

The targets that can potentially rule out common contamination of positive blood cultures include B. subtilis group, *Corynebacterium*, *C. acnes*, *Lactobacillus*, CoNS, and *Micrococcus*. The negative percent agreement for the clinical samples was high for all five targets (99.8 to 100%). The positive percent agreement prior to resolution of any discordant results for the clinical samples was >88% overall, with the exception of *Corynebacterium* spp. (78.4%). This was likely due to the variable SOC methods used by participating laboratories that may not adequately distinguish *Corynebacterium* spp. from other pleomorphic Gram-positive rods, such as, for example, *Brevibacterium* spp. Table S5 shows the 15 different *Corynebacterium* spp. detected by SOC methods. Most of the missed identifications were organisms identified by SOC to the genus level only.

Also, unique to this panel are the Pan targets. These are designed to detect polymicrobial infections, which in this study constituted 14.2% of the clinical samples. Occasionally, in mixed infections, a more slowly growing pathogen, such as a yeast, may not be seen on initial Gram stain when a more rapidly growing pathogen signals the blood culture instrument (K.C.C., personal observations). These Pan targets will alert the laboratory to a potential second organism that may be important in prompting additional diagnostic tests and possibly in continuing broad-based empirical therapy or adding antifungal therapy if it was not included in the empirical regimen. Given that Gram stain errors can vary to a certain degree by the site or reader, the Pan targets can also aid in cases where a Gram stain is misread or a Gram-variable organism may be present (64). Both the Pan *Candida* and Pan Gram-Negative targets performed well for the on-panel organisms in the clinical samples (PPA range, 92.4% to 95.7%, NPA range, 99.5% to 99.9%). Using ePlex RUO BCID panels, Huang et al. noted 100% specificity for the Pan Gram-Negative targets and one false negative, an E. coli isolate that was not detected out of a total of six samples that had a Gram-negative organism (34).

However, the BCID-GP Panel detected all organisms identified by comparative methods in 73% of polymicrobial samples. Accurate identification of all organisms in polymicrobial blood cultures remains a challenge for both molecular and standard-of-care methods, as demonstrated in this study, in which comparative methods had similar false-positivity and false-negativity rates. The variable performance seen in this study with polymicrobial infections is in line with that reported for studies of other positive blood culture syndromic panel tests (65–67).

The GenMark Dx ePlex system utilizes three panels for identification of Gram-positive, Gram-negative, and fungal organisms, all of which are now commercially available. The use of multiple panels allows for inclusion of a wider range of targets, but the selection of the appropriate panel or panels depends upon accurate Gram stain results. There were 1,220 clinical samples with only positive organisms seen on Gram stain. Of these, only 5 had Gram-negative bacteria or *Candida* species identified by the SOC methods. In addition, there were six samples in which the BCID-GP Panel Pan Gram-Negative or Pan *Candida* target detected an organism missed by the Gram stain and the SOC methods (data not shown). Overall, the positive predictive value of the Gram-positive stain was 99.1% for prediction of BCID-GP Panel selection in this study. This is consistent with overall accuracy of the interpretation of Gram stains from positive blood cultures of 99.3% reported by Rand and Tillan (68).

There are several limitations to this study. Because of the broad range of this panel and the low prevalence of some of the targeted organisms, the clinically collected samples had to be supplemented with a total of 566 contrived samples, and therefore, the results may be more favorable than in actual clinical practice. Also, retrospective clinical samples were frozen for up to 4 years before testing, which may have affected recovery of some of the more fastidious organisms. Though two sites used bioMérieux BacTAlert bottles for blood culture collection, this bottle type accounted for only 12% of the bottles collected; most sites used BD Bactec bottles. There were false positives that could not be resolved by comparator methods. There are several possible reasons for this observation, such as detection of nonviable organisms, as has been observed in other studies of molecular assays or contamination. Finally, biological contamination due to presence of nucleic acid from an organism introduced during manufacturing of the blood culture bottle broth has been reported (69). With respect to this, there were no observed patterns of repeated detection of an organism that was not seen on Gram stain.

In conclusion, the ePlex BCID-GP Panel compares favorably to SOC and targeted molecular methods and provides results much faster and with minimal hands-on time. It detects a broader range of pathogens to the genus and species levels than do other commercially available BCID panels, and it includes the antimicrobial resistance gene *mecC* and potential blood culture contaminants. The Pan Gram-Negative and Pan *Candida* targets are unique features of the panel that alert the user to the presence of mixed cultures that may not be detected by the Gram stain, assist in scenarios of misread Gram stains, and can further delineate what species might be present in the case of Gram variability. The targets representing potential contamination have the ability to reduce inappropriate antimicrobial utilization and reduce hospital costs associated with identification of contaminants.

## Supplementary Material

Supplemental file 1
