# Supplementary material for: Clinical Performance of the Novel GenMark Dx ePlex Blood Culture ID Gram-Positive Panel
Source: J Clin Microbiol. 2020 Mar 25;58(4):e01730-19. doi: 10.1128/JCM.01730-19 (PMC7098771; doi:10.1128/JCM.01730-19)
Supplement: Supplemental file 1 [file JCM.01730-19-s0001.pdf]

Supplemental Tables

Supplemental Table 1: Contrived Sample Organism Strain Frequency			
**Gram-Positive Organism**	**Strain**	**# Tested**	**CFU/Bottle (min-max)**
Bacillus amyloliquefaciens	ATCC23350	3	1.470E+05 - 1.770E+06
Bacillus amyloliquefaciens	ATCC23845	4	1.620E+05 - 1.680E+06
Bacillus amyloliquefaciens	ATCC53495	3	1.410E+05 - 1.590E+06
Bacillus atrophaeus	ATCC51189	4	1.590E+06 - 1.860E+06
Bacillus atrophaeus	ATCC6455	3	1.950E+05 - 1.650E+06
Bacillus atrophaeus	ATCC6537	4	1.620E+05 - 1.770E+06
Bacillus cereus	ATCC10876	11	1.560E+03 - 2.520E+05
Bacillus cereus	ATCC21769	10	1.440E+05 - 1.980E+05
Bacillus cereus	ATCC31430	9	1.800E+03 - 1.920E+05
Bacillus cereus	ATCC53522	10	1.290E+03 - 1.890E+05
Bacillus licheniformis	ATCC21039	3	1.470E+05 - 1.590E+06
Bacillus licheniformis	ATCC21667	3	1.410E+05 - 1.590E+06
Bacillus licheniformis	ATCC53926	4	1.470E+05 - 1.860E+06
Bacillus subtilis	ATCC15040	5	1.650E+05 - 1.620E+06
Bacillus subtilis	ATCC15561	8	1.740E+05 - 1.920E+06
Bacillus subtilis	ATCC55614	6	1.560E+05 - 1.650E+06
Bacillus thuringiensis	ATCC10792	2	1.470E+05 - 1.590E+06
Bacillus thuringiensis	ATCC33679	1	1.560E+05
Bacillus thuringiensis	ATCC55173	3	1.860E+03 - 1.920E+06
Corynebacterium coyleae	ATCC700219	7	1.710E+04 - 1.830E+04
Corynebacterium falsenii	ATCCBAA-596	9	1.650E+04 - 1.950E+04
Corynebacterium striatum	ATCCBAA-1293	4	2.040E+04
Enterococcus faecalis, vanA	JMI876745	10	1.620E+03 - 1.620E+04
Enterococcus faecalis, vanB	ATCC51299	11	1.560E+03 - 1.890E+05

Enterococcus faecalis, vanB	ATCC51575	11	1.410E+03 - 1.980E+05
Enterococcus faecalis, vanB	ATCC700802	10	1.560E+03 - 2.220E+05
Enterococcus faecalis, vanB	ATCCBAA-2365	10	1.590E+03 - 1.980E+05
Enterococcus faecium, vanA	ATCC51559	4	1.530E+05 - 2.040E+05
Enterococcus faecium, vanA	ATCC700221	3	1.740E+05 - 2.040E+05
Enterococcus faecium, vanA	ATCCBAA-2316	5	1.560E+05 - 1.920E+05
Enterococcus faecium, vanA	ATCCBAA-2317	3	1.590E+05 - 1.770E+05
Enterococcus faecium, vanA	ATCCBAA-2318	5	1.500E+03 - 1.920E+05
Enterococcus faecium, vanA	ATCCBAA-2319	5	1.470E+03 - 1.980E+05
Enterococcus faecium, vanA	ATCCBAA-2320	3	1.560E+05 - 1.980E+05
Enterococcus faecium, vanA	LMC002867	3	1.620E+05 - 1.980E+05
Enterococcus faecium, vanA	LMC003921	4	1.440E+05 - 2.070E+05
Enterococcus faecium, vanA	LMC032261	4	1.590E+05 - 1.890E+05
Enterococcus faecium, vanA	LMC055971	3	1.590E+05 - 1.680E+05
Enterococcus faecium, vanA	LMC103676	5	1.530E+05 - 1.860E+05
Enterococcus faecium, vanA	LMC104266	3	1.230E+05 - 1.740E+05
Enterococcus faecium, vanB	ATCC51858	10	1.500E+05 - 2.760E+05
Enterococcus flavescens	ATCC49996	3	1.200E+05 - 1.650E+05
Enterococcus gallinarum	ATCC49610	1	1.710E+05
Enterococcus gallinarum	ATCC700425	3	1.260E+05 - 1.620E+05
Enterococcus hirae	ATCC10541	1	1.860E+05
Enterococcus malodoratus	ATCC43197	3	1.290E+05 - 1.680E+05
Enterococcus raffinosus	ATCC49464	2	1.440E+05 - 1.590E+05
Enterococcus saccharolyticus	ATCC43076	1	1.320E+05
Lactobacillus casei	ATCC25598	2	1.770E+05 - 1.830E+05
Lactobacillus casei	ATCC334	6	1.050E+05 - 2.070E+05
Lactobacillus casei	ATCC39392	4	1.260E+05 - 1.680E+05
Lactobacillus paracasei	148-260	3	1.440E+05 - 1.800E+05
Lactobacillus paracasei	ATCC27092	2	1.350E+05 - 1.680E+05

Lactobacillus paracasei	ATCCBAA-52	6	1.560E+05 - 1.800E+05
Lactobacillus rhamnosus	ATCC39595	3	1.380E+05 - 1.620E+05
Lactobacillus rhamnosus	ATCC53103	5	1.500E+05 - 1.650E+05
Lactobacillus rhamnosus	ATCC55915	2	1.710E+05 - 2.640E+05
Listeria innocua	ATCC33090	4	1.500E+05 - 1.830E+05
Listeria innocua	NCTC11288	5	1.380E+05 - 2.040E+05
Listeria ivanovii	ATCC19119	2	1.380E+05 - 1.680E+05
Listeria ivanovii	ATCC700402	4	1.080E+05 - 1.800E+05
Listeria ivanovii	ATCCBAA-139	4	1.170E+05 - 1.860E+05
Listeria monocytogenes	ATCC13932	5	1.260E+05 - 1.830E+05
Listeria monocytogenes	ATCC19111	3	1.260E+05 - 1.770E+05
Listeria monocytogenes	ATCC19112	4	1.440E+05 - 1.830E+05
Listeria monocytogenes	ATCC19114	5	1.290E+05 - 1.650E+05
Listeria monocytogenes	ATCC19116	5	1.410E+05 - 1.710E+05
Listeria monocytogenes	ATCC19117	5	1.290E+05 - 1.830E+05
Listeria monocytogenes	ATCC19118	5	1.380E+05 - 2.760E+05
Listeria monocytogenes	ATCC7644	5	1.380E+05 - 1.800E+05
Listeria monocytogenes	ATCCBAA-751	5	1.230E+05 - 2.010E+05
Listeria monocytogenes	NCTC10890	4	1.380E+05 - 1.800E+05
Listeria seeligeri	ATCC35967	5	9.600E+04 - 1.830E+05
Listeria welshimeri	ATCC35897	5	1.380E+05 - 1.890E+05
Micrococcus luteus	ATCC10240	3	N/A
Micrococcus luteus	ATCC19212	3	N/A
Micrococcus luteus	ATCC400	3	N/A
Micrococcus luteus	ATCC4698	3	N/A
Micrococcus luteus	ATCC49732	3	N/A
Micrococcus luteus	ATCC53598	4	N/A
Micrococcus lylae	ATCC27566	4	N/A
Micrococcus yunnanensis	ATCC7468	4	N/A

Propionibacterium acnes	ATCC11827	8	1.350E+05 - 1.020E+06
Propionibacterium acnes	ATCC11828	6	1.320E+05 - 1.500E+06
Propionibacterium acnes	ATCC33179	4	1.140E+05 - 1.620E+06
Propionibacterium acnes	ATCC6919	8	1.380E+05 - 8.400E+05
Staphylococcus aureus, mecA	ATCC33591	3	1.620E+05 - 2.010E+05
Staphylococcus aureus, mecA	ATCCBAA-44	5	1.530E+05 - 1.680E+05
Staphylococcus aureus, mecA	NCTC12493	2	1.590E+05 - 1.680E+05
Staphylococcus aureus, mecC	ATCCBAA-2312	23	1.440E+03 - 1.950E+05
Staphylococcus aureus, mecC	ATCCBAA-2313	26	1.410E+03 - 2.940E+05
Staphylococcus epidermidis, mecA	ATCC35984	1	1.350E+05
Staphylococcus lugdunensis	ATCC49576	9	1.320E+05 - 2.040E+05
Staphylococcus lugdunensis	NRS878	9	1.440E+05 - 1.950E+05
Staphylococcus lugdunensis	NRS879	9	1.530E+05 - 1.980E+05
Staphylococcus lugdunensis	NRS880	9	9.000E+04 - 1.920E+05
Staphylococcus lugdunensis	NRS881	9	1.200E+05 - 1.950E+05
Streptococcus agalactiae	ATCC12403	2	1.680E+05 - 1.890E+05
Streptococcus agalactiae	ATCC12973	2	1.470E+05 - 1.920E+05
Streptococcus agalactiae	ATCC13813	2	1.650E+05 - 1.770E+05
Streptococcus agalactiae	ATCC27956	2	1.890E+05 - 1.980E+05
Streptococcus anginosus	ATCC700231	5	1.380E+05 - 1.890E+05
Streptococcus anginosus	ATCC9895	3	1.530E+05 - 1.650E+05
Streptococcus anginosus	NCTC10713	5	1.320E+05 - 1.890E+05
Streptococcus constellatus	ATCC27513	4	1.440E+05 - 1.890E+05
Streptococcus constellatus	ATCC27823	2	1.500E+05 - 1.920E+05
Streptococcus intermedius	ATCC27335	4	1.410E+05 - 1.590E+05
Streptococcus pyogenes	ATCC12344	5	1.710E+03 - 1.830E+05
Streptococcus pyogenes	ATCC12384	4	1.260E+05 - 1.830E+05
Streptococcus pyogenes	ATCC14289	4	1.710E+03 - 1.980E+05
Streptococcus pyogenes	ATCC19615	4	1.380E+03 - 1.710E+05

Streptococcus pyogenes	ATCC49399	5	1.740E+03 - 1.980E+05
Streptococcus pyogenes	NCIMB13285	4	1.560E+03 - 1.380E+05

Supplemental Table 2: Subject Demographics by Collection Type

	**Prospectively-Collected (N=711)**	**Retrospectively-Collected (N=586)**
Sex		
Male	377 (53.0)	317 (54.1)
Female	334 (47.0)	269 (45.9)
Age (years)		
N	711	584
Mean	54.7	57.8
Median	58.0	61.0
SD	23.8	21.2
Minimum	0.0	0.0
Maximum	90.0	90.0
<1 yr	27 (3.8)	11 (1.9)
1-17 yrs	42 (5.9)	17 (2.9)
18-44 yrs	121 (17.0)	104 (17.7)
45-64 yrs	250 (35.2)	193 (32.9)
65-84 yrs	217 (30.5)	209 (35.7)
85+ yrs	54 (7.6)	50 (8.5)
Unknown	---	2 (0.3)

**Supplemental Table 3. Positivity Rate (%) for the BCID-GP Panel by Age Group (Prospective Samples)**							
**Target**	**All Ages (N=711)**	**Age <1 (N=27)**	**Age 1-17 (N=42)**	**Age 18-44 (N=121)**	**Age 45-64 (N=250)**	**Age 65-84 (N=217)**	**Age 85+ (N=54)**
Bacillus cereus group	5 (0.7)	0 (0.0)	0 (0.0)	3 (2.5)	2 (0.8)	0 (0.0)	0 (0.0)
Bacillus subtilis group	2 (0.3)	0 (0.0)	0 (0.0)	1 (0.8)	0 (0.0)	0 (0.0)	1 (1.9)
Corynebacterium	14 (2.0)	1 (3.7)	0 (0.0)	4 (3.3)	5 (2.0)	4 (1.8)	0 (0.0)
Cutibacterium acnes (*P. acnes*)	8 (1.1)	0 (0.0)	0 (0.0)	3 (2.5)	2 (0.8)	3 (1.4)	0 (0.0)
Enterococcus	62 (8.7)	0 (0.0)	6 (14.3)	8 (6.6)	20 (8.0)	24 (11.1)	4 (7.4)
Enterococcus faecalis	50 (7.0)	0 (0.0)	6 (14.3)	6 (5.0)	15 (6.0)	20 (9.2)	3 (5.6)
Enterococcus faecium	12 (1.7)	0 (0.0)	0 (0.0)	1 (0.8)	6 (2.4)	5 (2.3)	0 (0.0)
Lactobacillus	5 (0.7)	0 (0.0)	0 (0.0)	2 (1.7)	1 (0.4)	1 (0.5)	1 (1.9)
Listeria	1 (0.1)	0 (0.0)	0 (0.0)	0 (0.0)	0 (0.0)	1 (0.5)	0 (0.0)
Listeria monocytogenes	0 (0.0)	0 (0.0)	0 (0.0)	0 (0.0)	0 (0.0)	0 (0.0)	0 (0.0)

Micrococcus	19 (2.7)	0 (0.0)	3 (7.1)	2 (1.7)	8 (3.2)	5 (2.3)	1 (1.9)
Staphylococcus	452 (63.6)	23 (85.2)	23 (54.8)	78 (64.5)	154 (61.6)	139 (64.1)	35 (64.8)
Staphylococcus aureus	162 (22.8)	8 (29.6)	4 (9.5)	37 (30.6)	69 (27.6)	38 (17.5)	6 (11.1)
Staphylococcus epidermidis	182 (25.6)	10 (37.0)	11 (26.2)	26 (21.5)	54 (21.6)	62 (28.6)	19 (35.2)
Staphylococcus lugdunensis	5 (0.7)	1 (3.7)	0 (0.0)	0 (0.0)	3 (1.2)	0 (0.0)	1 (1.9)
Streptococcus	110 (15.5)	5 (18.5)	9 (21.4)	16 (13.2)	40 (16.0)	31 (14.3)	9 (16.7)
Streptococcus agalactiae	12 (1.7)	1 (3.7)	0 (0.0)	1 (0.8)	5 (2.0)	5 (2.3)	0 (0.0)
Streptococcus anginosus group	10 (1.4)	0 (0.0)	0 (0.0)	2 (1.7)	3 (1.2)	3 (1.4)	2 (3.7)
Streptococcus pneumoniae	28 (3.9)	2 (7.4)	2 (4.8)	3 (2.5)	12 (4.8)	8 (3.7)	1 (1.9)
Streptococcus pyogenes	8 (1.1)	0 (0.0)	0 (0.0)	1 (0.8)	5 (2.0)	2 (0.9)	0 (0.0)
Pan *Candida*	0 (0.0)	0 (0.0)	0 (0.0)	0 (0.0)	0 (0.0)	0 (0.0)	0 (0.0)
Pan Gram-Negative	25 (3.5)	0 (0.0)	4 (9.5)	4 (3.3)	10 (4.0)	6 (2.8)	1 (1.9)
mecA (*Staphylococcus*)	261 (36.7)	14 (51.9)	10 (23.8)	41 (33.9)	83 (33.2)	94 (43.3)	19 (35.2)
mecA (*S. aureus*)	86 (12.1)	4 (14.8)	1 (2.4)	17 (14.0)	35 (14.0)	26 (12.0)	3 (5.6)
mecA (*S. epidermidis*)	137 (19.3)	8 (29.6)	9 (21.4)	19 (15.7)	38 (15.2)	48 (22.1)	15 (27.8)
mecA (*S. lugdunensis*)	1 (0.1)	0 (0.0)	0 (0.0)	0 (0.0)	1 (0.4)	0 (0.0)	0 (0.0)

mecA (CoNS excluding (*S. epidermidis/ S. lugdunensis*)	40 (5.6)	2 (7.4)	0 (0.0)	6 (5.0)	10 (4.0)	20 (9.2)	2 (3.7)
mecC (*Staphylococcus*)	0 (0.0)	0 (0.0)	0 (0.0)	0 (0.0)	0 (0.0)	0 (0.0)	0 (0.0)
vanA (*Enterococcus*)	9 (1.3)	0 (0.0)	0 (0.0)	0 (0.0)	6 (2.4)	3 (1.4)	0 (0.0)
vanA (*E. faecalis*)	1 (0.1)	0 (0.0)	0 (0.0)	0 (0.0)	1 (0.4)	0 (0.0)	0 (0.0)
vanA (*E. faecium*)	8 (1.1)	0 (0.0)	0 (0.0)	0 (0.0)	5 (2.0)	3 (1.4)	0 (0.0)
vanB (*Enterococcus*)	0 (0.0)	0 (0.0)	0 (0.0)	0 (0.0)	0 (0.0)	0 (0.0)	0 (0.0)

Supplemental Table 4. Organisms Identified by Standard of Care Methods not Targeted by the ePlex Panel

Off-Panel Organism Identified by SOC Organism	Count Number
Aerococcus sanguinicola	1
Aerococcus viridans	2
Bifidobacterium spp	1
Clostridium spp	2
Clostridium innocuum	1
Clostridium perfringens	2
Clostridium subterminale	1
Dermacoccus species	1
Globicatella sanguinis	1
Granulicatella adiacens	1
Lactococcus lactis	1
Microbacterium spp	1
Microbacterium/Cellulomonas	1
Mycobacterium fortuitum	1
Pediococcus acidilactici	1
Peptostreptococcus spp	2
Rothia spp	2
Rothia dentocariosa	2
Rothia mucilaginosa	2
No further identification – Aerobic gram-positive bacilli	1
No further identification - Anaerobic gram-positive cocci	1
TOTAL	28

**Supplemental Table 5. Clinical Performance of Group/Genus Gram Positive Targets in Species Detected by Comparator Methods or Contrived Species**		
	**Sensitivity/PPA**	
	**TP/TP+FN**	**% (95% CI)**
Bacillus cereus group	57/58	98.3 (90.9-99.7)
Bacillus cereus	49/50	98.0 (89.5-99.6)
Bacillus thuringiensis	8/8	100 (67.6-100)
Bacillus subtilis group	52/52	100 (93.1-100)
Bacillus amyloliquefaciens	11/11	100 (74.1-100)
Bacillus atrophaeus	11/11	100 (74.1-100)
Bacillus licheniformis	10/10	100 (72.2-100)
Bacillus subtilis	20/20	100 (83.9-100)
Corynebacterium	60/71	84.5 (74.3-91.1)
Corynebacterium	9/16	56.3 (33.2-76.9)
Corynebacterium afermentans	3/4	75.0 (30.1-95.4)
Corynebacterium amycolatum	1/1	100 (20.7-100)
Corynebacterium aurimucosum	2/2	100 (34.2-100)
Corynebacterium casei	1/1	100 (20.7-100)
Corynebacterium coyleae	10/10	100 (72.2-100)
Corynebacterium falsenii	9/9	100 (70.1-100)
Corynebacterium imitans	4/4	100 (51.0-100)
Corynebacterium jeikeium	4/5	80.0 (37.6-96.4)
Corynebacterium kroppenstedtii	1/1	100 (20.7-100)
Corynebacterium matruchotii	1/1	100 (20.7-100)
Corynebacterium mucifaciens	3/3	100 (43.9-100)
Corynebacterium pseudotuberculosis	0/1	0.0 (0.0-79.3)
Corynebacterium striatum	11/11	100 (74.1-100)
Corynebacterium tuberculostearicum	1/1	100 (20.7-100)
Corynebacterium urealyticum	0/1	0.0 (0.0-79.3)
Enterococcus	326/334	97.6 (95.3-98.8)

Enterococcus	1/1	100 (20.7-100)
Enterococcus avium	3/4	75.0 (30.1-95.4)
Enterococcus casseliflavus	0/1	0.0 (0.0-79.3)
Enterococcus casseliflavus/gallinarum	1/1	100 (20.7-100)
Enterococcus faecalis	186/191	97.4 (94.0-98.9)
Enterococcus faecium	124/125	99.2 (95.6-99.9)
Enterococcus flavescens	3/3	100 (43.9-100)
Enterococcus gallinarum	6/6	100 (61.0-100)
Enterococcus hirae	1/1	100 (20.7-100)
Enterococcus malodoratus	3/3	100 (43.9-100)
Enterococcus raffinosus	2/2	100 (34.2-100)
Enterococcus saccharolyticus	1/1	100 (20.7-100)
Lactobacillus	45/46	97.8 (88.7-99.6)
Lactobacillus casei	13/13	100 (77.2-100)
Lactobacillus paracasei	12/12	100 (75.8-100)
Lactobacillus rhamnosus	19/20	95.0 (76.4-99.1)
Lactobacillus zeae	1/1	100 (20.7-100)
Listeria	76/77	98.7 (93.0-99.8)
Listeria innocua	9/9	100 (70.1-100)
Listeria ivanovii	9/10	90.0 (59.6-98.2)
Listeria monocytogenes	48/48	100 (92.6-100)
Listeria seeligeri	5/5	100 (56.6-100)
Listeria welshimeri	5/5	100 (56.6-100)
Micrococcus	66/71	93.0 (84.6-97.0)
Micrococcus	18/22	81.8 (61.5-92.7)
Micrococcus luteus	36/36	100 (90.4-100)
Micrococcus luteus/lylae	4/5	80.0 (37.6-96.4)
Micrococcus lylae	4/4	100 (51.0-100)
Micrococcus yunnanensis	4/4	100 (51.0-100)
Staphylococcus	737/752	98.0 (96.7-98.8)
Coagulase-negative *Staphylococcus* (CoNS)	18/18	100 (82.4-100)

CoNS (not *S. epidermidis/lugdunensis*)	2/2	100 (34.2-100)
Staphylococcus	75/81	92.6 (84.8-96.6)
Staphylococcus aureus	338/342	98.8 (97.0-99.5)
Staphylococcus aureus ssp *aureus*	8/8	100 (67.6-100)
Staphylococcus auricularis	4/4	100 (51.0-100)
Staphylococcus capitis	21/21	100 (84.5-100)
Staphylococcus carnosus ssp *carnosus*	0/1	0.0 (0.0-79.3)
Staphylococcus cohnii	1/2	50.0 (9.5-90.5)
Staphylococcus epidermidis	155/160	96.9 (92.9-98.7)
Staphylococcus haemolyticus	8/8	100 (67.6-100)
Staphylococcus hominis	37/37	100 (90.6-100)
Staphylococcus hominis ssp *hominis*	27/27	100 (87.5-100)
Staphylococcus hominis ssp *novobiosepticus*	1/1	100 (20.7-100)
Staphylococcus lugdunensis	51/51	100 (93.0-100)
Staphylococcus pettenkoferi	2/2	100 (34.2-100)
Staphylococcus saccharolyticus	1/1	100 (20.7-100)
Staphylococcus saprophyticus	2/2	100 (34.2-100)
Staphylococcus schleiferi	1/1	100 (20.7-100)
Staphylococcus simulans	3/3	100 (43.9-100)
Staphylococcus warneri	4/4	100 (51.0-100)
Streptococcus	331/340	97.4 (95.0-98.6)
Alpha Hemolytic *Streptococcus*	1/1	100 (20.7-100)
Gamma Hemolytic *Streptococcus*	1/1	100 (20.7-100)
Streptococcus	5/7	71.4 (35.9-91.8)
Streptococcus - *viridans* group	16/19	84.2 (62.4-94.5)
Streptococcus Group G	1/1	100 (20.7-100)
Streptococcus agalactiae	55/56	98.2 (90.6-99.7)
Streptococcus anginosus	27/27	100 (87.5-100)
Streptococcus anginosus group	26/26	100 (87.1-100)
Streptococcus bovis	2/2	100 (34.2-100)
Streptococcus bovis group	1/1	100 (20.7-100)
Streptococcus constellatus	6/6	100 (61.0-100)

Streptococcus constellatus ssp *constellatus*	2/2	100 (34.2-100)
Streptococcus constellatus ssp *pharyngis*	1/1	100 (20.7-100)
Streptococcus dysgalactiae	2/2	100 (34.2-100)
Streptococcus dysgalactiae (Group G)	5/5	100 (56.6-100)
Streptococcus gallolyticus	1/1	100 (20.7-100)
Streptococcus gordonii	1/1	100 (20.7-100)
Streptococcus infantarius	1/1	100 (20.7-100)
Streptococcus intermedius	6/6	100 (61.0-100)
Streptococcus mitis	23/25	92.0 (75.0-97.8)
Streptococcus mitis group	10/10	100 (72.2-100)
Streptococcus mutans	1/1	100 (20.7-100)
Streptococcus oralis	3/3	100 (43.9-100)
Streptococcus parasanguinis	6/6	100 (61.0-100)
Streptococcus pneumoniae	69/69	100 (94.7-100)
Streptococcus pyogenes	53/54	98.1 (90.2-99.7)
Streptococcus salivarius	9/9	100 (70.1-100)
Streptococcus vestibularis	1/1	100 (20.7-100)
Streptococcus anginosus group	65/68	95.6 (87.8-98.5)
Streptococcus anginosus	25/27	92.6 (76.6-97.9)
Streptococcus anginosus group	25/26	96.2 (81.1-99.3)
Streptococcus constellatus	6/6	100 (61.0-100)
Streptococcus constellatus ssp *constellatus*	2/2	100 (34.2-100)
Streptococcus constellatus ssp *pharyngis*	1/1	100 (20.7-100)
Streptococcus intermedius	6/6	100 (61.0-100)

Supplemental Table 6: BCID-GP Panel Clinical Performance Post Discordant Resolution

	Target	Combined Samples			
		Sensitivity/PPA		Specificity/NPA	
		TP/TP+FN	% (95% CI)	TN/TN+FP	% (95% CI)
**Bacterial Pathogen Targets**	*B. cereus* group	57/58	98.3 (90.9-99.7)	1804/1804	100 (99.8-100)
	Enterococcus	327/334	97.9 (95.7-99.0)	1528/1528	100 (99.7-100)
	E. faecalis	183/187	97.9 (94.6-99.2)	1674/1674	100 (99.8-100)
	E. faecium	128/130	98.5 (94.6-99.6)	1728/1731	99.8 (99.5-99.9)
	Listeria	76/77	98.7 (93.0-99.8)	1784/1785	99.9 (99.7-100)
	L. monocytogenes	48/48	100 (92.6-100)	1814/1814	100 (99.8-100)
	Staphylococcus	746/756	98.7 (97.6-99.3)	1105/1106	99.9 (99.5-100)
	S. aureus	346/352	98.3 (96.3-99.2)	1429/1430	99.9 (99.6-100)
	S. epidermidis	152/156	97.4 (93.6-99.0)	1591/1607	99.0 (98.4-99.4)
	S. lugdunensis	53/53	100 (93.2-100)	1709/1710	99.9 (99.7-100)
	Streptococcus	339/344	98.5 (96.6-99.4)	1517/1518	99.9 (99.6-100)
	S. agalactiae	55/55	100 (93.5-100)	1779/1780	99.9 (99.7-100)
	S. anginosus grp	66/66	100 (94.5-100)	1767/1769	99.9 (99.6-100)
	S. pneumoniae	67/67	100 (94.6-100)	1766/1767	99.9 (99.7-100)
	S. pyogenes	53/54	98.1 (90.2-99.7)	1780/1780	100 (99.8-100)
**Pan Targets**	Pan *Candida*	98/105	93.3 (86.9-96.7)	2236/2237	100 (99.7-100)
	Pan Gram-Negative	424/442	95.9 (93.7-97.4)	1895/1900	99.7 (99.4-99.9)
**Antimicrobial Resistance Gene Targets**	*mecA*	416/426	97.7 (95.7-98.7)	319/326	97.9 (95.6-99.0)
	mecC	49/49	100 (92.7-100)	703/703	100 (99.5-100)
	vanA	122/124	98.4 (94.3-99.6)	209/210	99.5 (97.4-99.9)
	vanB	53/53	100 (93.2-100)	281/281	100 (98.7-100)
**Contamination Rule-Out Targets**	*B. subtilis* group	52/52	100 (93.1-100)	1809/1809	100 (99.8-100)
	Corynebacterium	62/68	91.2 (82.1-95.9)	1794/1794	100 (99.8-100)
	Cutibacterium acnes	44/47	93.6 (82.8-97.8)	1814/1815	99.9 (99.7-100)
	Lactobacillus	46/47	97.9 (88.9-99.6)	1814/1815	99.9 (99.7-100)
	Micrococcus	66/68	97.1 (89.9-99.2)	1793/1794	99.9 (99.7-100)

Supplemental Table 7. Monomicrobial Infections With Discordant Results (BCID-GP False Negative or False Positive vs Comparator Methods)

Comparator Methods			BCID-GP Panel	
**Organisms**	**ARG**	**No. of Samples**	**FN (No.)**	**FP (No.)**
		1		***Corynebacterium* (1)**
		1		***S. agalactiae* (1)**
		1		***Staphylococcus* (1)**
		2		***Streptococcus* (2)**
Bacillus cereus		1	*B. cereus* (1)	
Campylobacter gracilis		1	*C. gracilis* (1)	
Corynebacterium		4	*Corynebacterium* (4)	
Corynebacterium afermentans		1	***C. afermentans* (1)**	
Corynebacterium jeikeium		1	*C. jeikeium* (1)	
Corynebacterium pseudotuberculosis		1	***C. pseudotuberculosis* (1)**	
Corynebacterium		1	***Corynebacterium* (1)**	
E. faecalis	*vanA*	1	***vanA* (1)**	
E. faecium		1		*vanA* (1)
E. faecium		1		***vanA* (1)**
Micrococcus		1	*Micrococcus* (1)	
Micrococcus luteus/lylae		1	*M. luteus/lylae* (1)	
Micrococcus		1	***Micrococcus* (1)**	
S. agalactiae		1	***S. agalactiae* (1)**	
S. anginosus gp		1	***S. anginosus* gp (1)**	
S. anginosus		1	***S. anginosus* (1)**	
S. aureus		1	*S. aureus* (1)	
S. aureus		1		*mecA* (1)
S. aureus		1		***mecA* (1)**
S. aureus	*mecA*	1	*mecA* (1)	
S. aureus		1	***S. aureus* (1)**	
S. epidermidis		1		*mecA* (1)
S. epidermidis		2		***mecA* (2)**
S. epidermidis	*mecA*	1	*mecA* (1)	
S. epidermidis		1	***S. epidermidis* (1)**	
S. epidermidis	*mecA*	1	***S. epidermidis* (1)**	

S. pneumoniae		2	***S. pneumoniae* (2)**	
Staphylococcus		3		*mecA* (3)
Staphylococcus	*mecA*	1	***mecA* (1)**	
Staphylococcus capitis	*mecA*	1	*mecA* (1)	
Staphylococcus carnosus ssp *carnosus*		1	***S. carnosus* (1)**	
Staphylococcus		1	***Staphylococcus* (1)**	
Streptococcus mitis		1	*S. mitis* (1)	
Streptococcus mitis		1	***S. mitis* (1)**	
Total		46	31	15
True Negative			17	
True Positive				9

ARG = Antimicrobial Resistance Gene, FP = False Positive, FN = False Negative
Bolded FN organisms were not detected by discordant resolution methods.
Bolded FP organisms were detected by discordant resolution methods.
Bolded FN resistance genes indicate the comparator result was contaminated and the resistance gene was not present.
Bolded FP resistance genes were detected by an FDA-cleared multiplex PCR assay.

Supplemental Table 8. Detections With Discordant Results (BCID-GP False Negative or False Positive vs Comparator Methods)							
Organisms/ARGs Identified by Comparator Methods						BCID-GP Panel	
Org 1	Org 2	Org 3	Org 4	ARG	No. of Samples	FN (No.)	FP (No.)
C. acnes					1	*C. acnes* (1)	***S. aureus* (1)**
Corynebacterium urealyticum					1	***C. urealyticum* (1)**	***Staphylococcus* (1)**
Corynebacterium					1	***Corynebacterium* (1)**	***C. acnes* (1)**
E. faecalis					3		*E. faecium* (3)
E. faecalis					1		***E. faecium* (1)**
E. faecalis				*vanA*	1		***E. faecium* (1)**
E. faecalis					1	***E. faecalis* (1)**	***E. faecium* (1)**
E. faecalis					1	***E. faecalis* (1)**	Pan *Candida* (1)
E. faecium				*vanA*	1		***Streptococcus* (1)**
Micrococcus					2	***Micrococcus* (2)**	***Staphylococcus* (2)**
Rothia species*					1		Pan GN (1)
S. agalactiae					1	***S. agalactiae* (1)**	*S. aureus* (1)
S. anginosus gp					1		*Staphylococcus* (1)
S. aureus				*mecA*	2		*S. epidermidis* (2)
S. aureus				*mecA*	1		***Streptococcus* (1)**
S. aureus					1	***S. aureus* (1)**	**Pan GN (1)**
S. aureus					1	***S. aureus* (1)**	*S. epidermidis* (1), *mecA* (1)
S. epidermidis				*mecA*	1		Pan GN (1)
S. epidermidis				*mecA*	1		***S. lugdunensis* (1)**
S. epidermidis					1	***S. epidermidis* (1)**	**Pan GN (1)**
S. epidermidis					1	***S. epidermidis* (1)**	*S. agalactiae* (1)
S. epidermidis					2	***S. epidermidis* (2)**	***S. aureus* (2)**
S. epidermidis				*mecA*	1	***S. epidermidis* (1)**	***S. aureus* (1)**
S. pneumoniae					1	***S. pneumoniae* (1)**	*S. anginosus* gp (1)
S. pyogenes					1		*Micrococcus* (1)
Staphylococcus					1		*S. anginosus* gp (1)
Staphylococcus capitis					1		*C. acnes* (1)
Staphylococcus capitis				*mecA*	2		*S. epidermidis* (2)

**Supplemental Table 8. Detections with Discordant Results (BCID-GP False Negative or False Positive vs Comparator Methods)**							
**Organisms/ARGs Identified by Comparator Methods**						**BCID-GP Panel**	
**Org 1**	**Org 2**	**Org 3**	**Org 4**	**ARG**	**No. of Samples**	**FN (No.)**	**FP (No.)**
Staphylococcus haemolyticus					1		***S. epidermidis*** **(1),** ***S. lugdunensis*** **(1)**
Staphylococcus haemolyticus				*mecA*	1		***S. epidermidis*** **(1)**
Staphylococcus haemolyticus				*mecA*	1		***Streptococcus*** **(1)**
Staphylococcus hominis					1		*Listeria* (1)
Staphylococcus hominis					1		*S. epidermidis* (1)
Staphylococcus hominis				*mecA*	3		*S. epidermidis* (3)
Staphylococcus hominis ssp *novobiosepticus*				*mecA*	1		*S. epidermidis* (1)
Staphylococcus					1	***Staphylococcus*** **(1)**	***Streptococcus*** **(1)**
Streptococcus					1		***S. epidermidis*** **(1)**
Streptococcus - viridans gp					1	***Streptococcus*** **(1)**	***Enterococcus*** **(1)**
Streptococcus - viridans gp					1	***Streptococcus*** **(1)**	***S. aureus*** **(1)**
Streptococcus mitis					1		***S. anginosus*** **gp (1)**
Streptococcus mitis					1		***S. pneumoniae*** **(1)**
A. baumannii	*E. faecalis*			*vanA*	2	Pan GN (2)	
Acinetobacter lwoffii^	*Staphylococcus hominis*			*mecA*	1		*S. epidermidis* (1)
C. acnes	*Enterococcus avium*			*vanA*	1	*E. avium* (1), ***vanA*** **(1)**	
C. acnes	*S. epidermidis*				1	*S. epidermidis* (1)	
C. acnes	*S. lugdunensis*				1	*C. acnes* (1)	
C. albicans^	*E. faecalis*			*vanA*	1	***E. faecalis*** **(1)**	***E. faecium*** **(1)**
C. glabrata	*Lactobacillus rhamnosus*				1	Pan *Candida* (1)	
C. parapsilosis	*E. faecalis*			*vanA*	1	Pan *Candida* (1)	***Staphylococcus*** **(1)**

Supplemental Table 8. Detections with Discordant Results (BCID-GP False Negative or False Positive vs Comparator Methods)

Organisms/ARGs Identified by Comparator Methods						BCID-GP Panel	
**Org 1**	**Org 2**	**Org 3**	**Org 4**	**ARG**	**No. of Samples**	**FN (No.)**	**FP (No.)**
Corynebacterium	*Streptococcus*				1	*Corynebacterium* (1)	
E. coli^	*S. aureus*			*mecA*	1		*S. epidermidis* (1)
E. faecalis	*E. faecium*				1	*E. faecium* (1)	
E. faecalis	*P. mirabilis*				1	Pan GN (1)	
E. faecalis	*P. mirabilis*^				1	*E. faecalis* (1)	
E. faecalis	*P. mirabilis*^			*vanA*	1	*E. faecalis* (1), *vanA* (1)	
E. faecalis	*Providencia stuartii*				1	Pan GN (1)	
E. faecalis	*S. aureus*			*mecA*	1	*S. aureus* (1), *mecA* (1)	
E. faecalis	*K. pneumoniae*			*vanA*	1	***E. faecalis* (1),** Pan GN (1)	***E. faecium* (1)**
E. faecium	*P. aeruginosa*^			*vanA*	1		*Lactobacillus* (1)
K. pneumoniae^	*S. aureus*				1	*S. aureus* (1)	
L. monocytogenes	*Staphylococcus*			*mecA*	1	*Staphylococcus* (1), ***mecA* (1)**	
Lactobacillus casei	*Veillonella* species				1	Pan GN (1)	
P. mirabilis^	*Staphylococcus*			*mecA*	1	*Staphylococcus* (1), *mecA* (1)	
*Rothia mucilaginosa**	*Streptococcus* - viridans gp				1		***Lactobacillus* (1)**
S. agalactiae	*S. aureus*				1	*S. aureus* (1)	Pan GN (1)
S. agalactiae	*S. aureus*			*mecA*	1		*S. epidermidis* (1)
S. anginosus gp	*S. aureus*				1		Pan GN (1)
S. anginosus	*Streptococcus mitis*				1	***S. anginosus gp* (1)**	
S. aureus	*S. epidermidis*				1	*S. aureus* (1)	***mecA* (1)**
S. aureus	*S. pyogenes*			*mecA*	1	*S. pyogenes* (1)	
S. epidermidis	*S. lugdunensis*			*mecA*	1		***Corynebacterium* (1)**
S. epidermidis	*Staphylococcus hominis*			*mecA*	1	*S. epidermidis* (1)	
S. epidermidis	*Streptococcus* - viridans gp				1	*S. epidermidis* (1)	
S. maltophilia^	*Streptococcus*				1	*Streptococcus* (1)	

Supplemental Table 8. Detections with Discordant Results (BCID-GP False Negative or False Positive vs Comparator Methods)							
Organisms/ARGs Identified by Comparator Methods						BCID-GP Panel	
**Org 1**	**Org 2**	**Org 3**	**Org 4**	**ARG**	**No. of Samples**	**FN (No.)**	**FP (No.)**
Staphylococcus capitis	*Staphylococcus hominis*			*mecA*	1		*S. epidermidis* (1)
Staphylococcus cohnii	*Streptococcus* - viridans gp				1	*Streptococcus* (1)	
Staphylococcus hominis	*Staphylococcus pettenkoferi*				1		*S. epidermidis* (1), *mecA* (1)
Staphylococcus hominis	*Streptococcus mitis*			*mecA*	1	*mecA* (1)	
*A. baumannii^*	*E. faecalis*	*S. aureus*		*mecA*	1	*mecA* (1)	
*C. albicans^*	*E. faecium*	*Staphylococcus hominis*		*mecA, vanA*	1		*S. epidermidis* (1)
*Citrobacter freundii^*	*K. pneumoniae^*	*Staphylococcus hominis*		*mecA*	1	*mecA* (1)	
Corynebacterium	*S. epidermidis*	*Streptococcus*		*mecA*	1	*Streptococcus* (1)	
E. faecalis	*M. morganii^*	*Proteus vulgaris^*		*vanA*	1	*E. faecalis* (1), *vanA* (1)	
E. faecalis	*P. aeruginosa*	*S. aureus*		*mecA*	1	*E. faecalis* (1), Pan GN (1)	
E. faecium	*S. epidermidis*	*Staphylococcus hominis*		*mecA*	1	*E. faecium* (1)	
S. agalactiae	*S. aureus*	*Staphylococcus*		*mecA*	1	*Staphylococcus* (1), *S. aureus* (1), *mecA* (1)	
S. marcescens	*Streptococcus mitis gp*$^{+}$	*Streptococcus salivarius*			1		*S. pneumoniae* (1)
*Aerococcus viridans**	*K. oxytoca^*	*S. epidermidis*	*Staphylococcus cohnii*	*mecA*	1	*Staphylococcus* (1), *S. epidermidis* (1), *mecA* (1)	
*Aeromonas caviae^*	*E. coli^*	*Enterococcus casseliflavus*	*K. oxytoca^*		1	*Enterococcus* (1)	
Lactobacillus rhamnosus	*S. anginosus* gp	*Staphylococcus*	*Streptococcus* - viridans gp		1	*Staphylococcus* (1)	

**Supplemental Table 8. Detections with Discordant Results (BCID-GP False Negative or False Positive vs Comparator Methods)**							
**Organisms/ARGs Identified by Comparator Methods**						**BCID-GP Panel**	
**Org 1**	**Org 2**	**Org 3**	**Org 4**	**ARG**	**No. of Samples**	**FN (No.)**	**FP (No.)**

Note:

In any instance where multiple species of a single genus are present, the BCID-GP Panel will detect the applicable genus call and any species level calls that are present on the panel. Multiple species may be rolled into the genus level call and would be represented by a single genus level call.

Nomenclature in the ePlex BCID-GP Panel false negative or false positive columns is representative of the corresponding target on the ePlex BCID-GP Panel.

*Off-panel organism not targeted by the BCID-GP Panel.

^The BCID-GP Panel detected this organism with a Pan target.

[+] Site's SOC method identified *S. mitis* group; PCR/sequencing identified the organism as *S. mitis* (blast 94.1%). The BCID-GP Panel detected *S. pneumoniae*, which was reported as a false positive result.

Bolded FN organisms were not detected by discordant resolution methods. Bolded FP organisms were detected by discordant resolution methods.

Bolded FN resistance genes indicate the comparator result was contaminated and the resistance gene was not present. Bolded FP resistance genes were detected by an FDA-cleared multiplex PCR assay.
